# Differential growth rate, water-use efficiency and climate sensitivity between males and females of *Ilex aquifolium* in north-western Spain

**DOI:** 10.1093/aob/mcae126

**Published:** 2024-08-07

**Authors:** Julia Sánchez Vilas, Héctor Hernández-Alonso, Vicente Rozas, Rubén Retuerto

**Affiliations:** Departamento de Bioloxía Funcional (Área de Ecoloxía), Facultade de Bioloxía, Universidade de Santiago de Compostela, c/ Lope Gómez de Marzoa s/n, 15782 Santiago de Compostela, Spain; School of Biosciences, Sir Martin Evans Building, Cardiff University, CF10 3AX Cardiff, UK; EiFAB, iuFOR, Universidad de Valladolid, Campus Duques de Soria, 42004 Soria, Spain; CEFE, Univ Montpellier, CNRS, EPHE, IRD, Montpellier, France; EiFAB, iuFOR, Universidad de Valladolid, Campus Duques de Soria, 42004 Soria, Spain; Departamento de Bioloxía Funcional (Área de Ecoloxía), Facultade de Bioloxía, Universidade de Santiago de Compostela, c/ Lope Gómez de Marzoa s/n, 15782 Santiago de Compostela, Spain

**Keywords:** Dendroecology, dioecy, sexual dimorphism, tree growth, carbon isotope discrimination, water-use efficiency

## Abstract

**Background and aims:**

Dioecious plant species, i.e. those in which male and female functions are housed in different individuals, are particularly vulnerable to global environmental changes. For long-lived plant species, such as trees, long-term studies are imperative to understand how growth patterns and their sensitivity to climate variability affect the sexes differentially.

**Methods:**

Here, we explore long-term intersexual differences in wood traits, namely radial growth rates and water-use efficiency quantified as stable carbon isotope abundance of wood cellulose, and their climate sensitivity in *Ilex aquifolium* trees growing in a natural population in north-western Spain.

**Key results:**

We found that sex differences in secondary growth rates were variable over time, with males outperforming females in both radial growth rates and water-use efficiency in recent decades. Summer water stress significantly reduced the growth of female trees in the following growing season, whereas the growth of male trees was favoured primarily by cloudy and rainy conditions in the previous autumn and winter combined with low cloud cover and warm conditions in summer. Sex-dependent lagged correlations between radial growth and water availability were found, with a strong association between tree growth and cumulative water availability in females at 30 months and in males at 10 months.

**Conclusions:**

Overall, our results point to greater vulnerability of female trees to increasing drought, which could lead to sex-ratio biases threatening population viability in the future.

## INTRODUCTION

Dioecy, in which separate male and female individuals coexist in a population, is a relatively rare sexual system in flowering plants, occurring in ~6 % of species (Renner, 2014). Nevertheless, dioecy has evolved independently many times and is widespread in angiosperms, occurring in almost half of all angiosperm families (Heilbuth, 2000; Vamosi *et al.*, 2003; Renner, 2014). Avoidance of self-fertilization, with the corresponding expression of inbreeding depression, and sexual specialization have been claimed as two broad reasons for the selection of separate sexes (for a review, see Pannell and Jordan, 2022). Once dioecy occurs, females and males are expected to differentiate and specialize according to their individual sexual roles and associated resource needs, which can result in differences in function and performance between the sexes (Obeso, 2002; Barrett and Hough, 2013). Sexual dimorphism can occur not only in the reproductive organs, but also in the morphology, physiology and life-history traits, which is also known as secondary sexual dimorphism (Obeso, 2002; Barrett and Hough, 2013). Such differences have been attributed to resource trade-offs between allocation to reproduction and to other functions, such as growth and maintenance (Obeso, 2002).

The general assumption is that females of dioecious woody plants usually expend proportionally more resources on reproduction because they produce not only flowers but also fruits (Obeso, 2002; Barrett and Hough, 2013), hence they are expected to grow less and perform worse under environmental stress (Juvany and Munné-Bosch, 2015). However, there is still no universal pattern, and some studies have observed equal or even greater reproductive effort in males and greater growth and performance in females (Barrett and Hough, 2013; Juvany and Munné-Bosch, 2015). It was found that the presence and direction of differences between males and females are species specific and highly context dependent (Retuerto *et al.*, 2000, 2018; Juvany and Munné-Bosch, 2015). Therefore, there is a need for further data, particularly on sex-related differences in physiology of dioecious woody angiosperms in response to environmental stress.

The physiological aspects have been less studied than morphological or growth aspects, probably owing to the association of dioecy with a set of life-history traits (e.g. size, woodiness, perenniality) that challenge physiological measurement, integration and generalization (Vamosi *et al.*, 2003). However, the physiological comparison between the sexes is of great relevance because compensatory mechanisms can play a role in mitigating reproductive costs, and differences in physiological performance can lead to sexual differences in growth, survival and even population structure (Retuerto *et al.*, 2006, 2018; Juvany and Munné-Bosch, 2015). Physiological comparison between the sexes has been studied primarily using leaf-scale instantaneous measurements, such as photosynthetic activity, transpiration rate and other water-relationship parameters (Dawson and Ehleringer, 1993; Dawson *et al.*, 2004). However, short-term studies or single time-point studies can be misleading because the sexes can differ in their developmental timing, including the timing of investment in reproductive allocation (Milla *et al.*, 2006; Sánchez Vilas and Pannell, 2011) and also in the frequency and intensity of reproductive events (Obeso, 2002). In this context, further research is needed to understand thoroughly and model realistically the quantitative relationships between male and female reproductive efforts over the entire growing season or life cycle, particularly in woody species. This aspect is particularly important in the current context of climate change, because if growth and physiological responses to factors such as rising air temperature, increasing drought and CO_2_ concentration differ between the sexes of dioecious species, this could make them more vulnerable to changes in sex ratios and potentially lead to population decline and eventual extinction (Tognetti, 2012; Petry *et al.*, 2016).

Dendroecological studies are promising for comparing wood traits of males and females by providing a holistic, comprehensive and long-term analysis of tree responses to environmental changes. Radial growth of the woody stem is a highly integrative trait that is useful for assessing long-term plant efficiency because it reflects the overall performance of metabolic processes (Eckes-Shephard *et al.*, 2022). In addition, stable carbon isotopes in tree rings provide time-integrated insights into ecophysiological processes related to plant water stress (Gessler *et al.*, 2014), such as leaf conductance, hydraulic capacity and photosynthetic capacity (Körner *et al.*, 1988; Farquhar *et al.*, 1989; Ehleringer, 1993), pre-dawn water potential (Damesin *et al.*, 1998; Warren *et al.*, 2001) and climatic gradients of air humidity (Saurer and Siegenthaler, 1989) or precipitation (Korol *et al.*, 1999; Warren *et al.*, 2001; Hartman and Danin, 2010). Carbon isotope discrimination (Δ^13^C), which reflects the degree of discrimination against the ^13^C isotope, is often used to quantify the water-use efficiency, i.e. the ratio of net photosynthesis to stomatal conductance to water vapour, integrated over time (Farquhar *et al.*, 1982; Seibt *et al.*, 2008). Differences in Δ^13^C between the sexes of dioecious plants have been reported (Dawson and Bliss, 1989; Dawson and Ehleringer, 1993; Retuerto *et al.*, 2000). To improve our understanding of the ecological meaning of Δ^13^C levels, Ferrio *et al.* (2003) have suggested supplementing Δ^13^C analyses with other indicators of plant function, such as morphological traits (Fleck *et al.*, 1996; Damesin *et al.*, 1997) or plant growth (Dupouey *et al.*, 1993; Brooks *et al.*, 1998).

The aim of this study was to examine long-term intersexual differences in wood traits, namely radial growth and stable carbon isotopes, and their climate sensitivity in the dioecious tree *Ilex aquifolium* (European holly) growing in a natural population in Galicia, north-western Spain. Specifically, we aimed to determine whether sexual differences in secondary growth rates are stable over time and to investigate whether there is a sex-dependent response of secondary growth to climatic conditions in male and female trees. Previous research identified significant differences in the physiological responses of male and female *I. aquifolium* trees to resource availability (Retuerto *et al.*, 2000), but efforts to contrast these results on a wide temporal scale and to assess possible sex-dependent effects on temporal stability remain unexplored. Therefore, we expect different growth rates, water-use efficiency and climate sensitivity between males and females of *I. aquifolium*.

## MATERIALS AND METHODS

### Study species

*Ilex aquifolium* (Aquifoliaceae), the European holly, is a broad-leaved evergreen tree or shrub ≤23 m tall that often grows in the understorey of temperate forests, such as in our study site, the Sierra de Ancares, a protected area in the north-west of Spain (see further details below). This species was chosen owing to its abundance in the study site and owing to its relevant ecological role as a persistent (evergreen) understorey providing important habitat and food to woodland birds and grazers (Pollo *et al.*, 2005). The natural range of *I. aquifolium* extends from north-western Europe (64°N), where it is restricted to coastal areas, through central and southern Europe to North Africa (Algeria and Tunisia) and Asia Minor (34°N), where it grows from sea level to mountainous areas (Peterken and Lloyd, 1967). Holly does not occur in areas where the average temperature in January is below −4.6 °C and the average temperature of the warmest month does not exceed 12 °C (De Candolle, 1855; Iversen, 1944). Depending on substrate requirements, holly grows in different soil types, from acidic podzols to chalk soils or limestone rocks (Peterken and Lloyd, 1967). The flowers, which are clustered axillary on shoots that emerged in the previous year, are dioecious or, very rarely, hermaphroditic (Ward, 1904). Individuals can produce flowers from May to June from the tenth year, almost never if the individuals are <1.5 m tall. The fruit is a scarlet spherical drupe (7–12 mm) containing four pyrenes and is typically dispersed by birds. Fruit production varies greatly from year to year (Guitián and Bermejo, 2006), but good fruiting years appear to correlate with the duration of sunshine in July and the air temperature of the previous year, in addition to the absence of severe late-spring frosts (Peterken and Lloyd, 1967). Fruits ripen in late autumn and usually last throughout the winter. It was reported that the reproductive allocation (the ratio of mass of sexual structures to branch mass) was 56 ± 87 % (mean ± 1 s.d.) of the branch dry mass in females at the time of fruiting and 7 ± 3 % in males at the time of flowering (Obeso, 1997). The wood is hard and heavy, with small vessels characteristically arranged in radial rows (Schweingruber and Baas, 2011).

### Study site

**Fig. 1.**
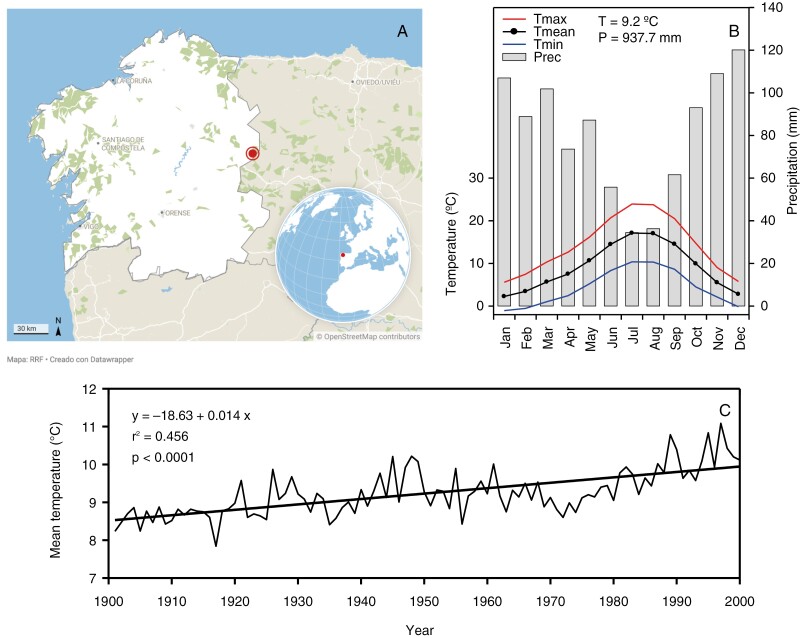
(A) Location of the study area in the Sierra de Ancares, Galicia, north-western Spain. (B) Climatic diagram of the Sierra de Ancares for the period 1901–2000. Maximum (Tmax), mean (Tmean) and minimum (Tmin) monthly temperatures and total monthly precipitation (Prec) series are displayed along with mean annual temperature (T) and total annual precipitation (P). (C) Temporal variation of the mean annual temperature in the period 1901–2000, with its linear fit, *r*^2^ and *P*-values are shown.

The study was conducted at the site of ‘Cavana Vella’ (Lugo, Galicia, north-western Spain), in the Sierra de Ancares, on the western edge of the Cantabrian Range. Sierra de Ancares is a protected area and was declared a UNESCO Biosphere Reserve in 2006. The studied area, located on a north-facing slope, was delimited by coordinates 6°52ʹ43″–6°54ʹ8″W (longitude) and 42°47ʹ52″–42°48ʹ2″N (latitude), with a mean elevation of 1400 m a.s.l. (Fig. 1A). The area belongs to the Eurosiberian biogeographical region but is very close to the Mediterranean region. Annual mean precipitation is 938 mm and annual mean temperature 9.2 °C (Fig. 1B). The mean temperature is −2.1 °C in the coldest month and 15.7 °C in the warmest month. An increasing trend in mean annual temperature was observed in the study area (Fig. 1C). The sampling site is an old-growth forest dominated by *I. aquifolium*, with a much lower density of other woody species (*Corylus avellana*, *Quercus petraea*, *Sorbus aucuparia*, *Betula alba* and *Taxus baccata*).

### Sampling procedure and tree-ring growth analysis

In July 2002, 70 mature, dominant and healthy *I. aquifolium* trees, 35 of each sex, were selected for sampling in the study area. Tree bole diameter was measured, and trees were cored to the pith at breast height using a Pressler increment borer. Three 5-mm-diameter wood cores were taken from each tree from southern, eastern and western exposures to facilitate cross-dating of the tree-ring series. The cores were dried for 3 weeks and polished with progressively finer sandpaper until the cellular structure of the xylem was clearly visible. The years corresponding to each ring were then identified, and the annual ring widths (in millimetres) were measured to the nearest 0.01 mm using a dendrochronometer (digital positiometer, Kütschenreiter, Austria), then cross-dated. Ring-width series were cross-dated to match similar relative ring-width patterns across years using distinctive ring features for different years that allowed mutual identification between trees. The quality of the cross-dating was checked using COFECHA software (Holmes, 1983), and possible dating errors in the tree-ring series were identified and corrected. Owing to difficulties in cross-dating, we were able to assign clearly rings with years in complete cores from only 16 male and 14 female trees (Table 1).

**Table 1.** Characteristics of the studied male and female *Ilex aquifolium* trees, individual secondary growth rates (BAI) and carbon isotope discrimination (∆^13^C) series from Cavana Vella, Sierra de Ancares, Lugo, Spain.

Characteristic	Male	Female
Number of trees	16	14
Number of cores	43	32
Stem diameter, cm, mean (±s.e.)	18.5 ± 0.7	18.5 ± 0.9
Stem diameter, cm, range	14.2–23.1	13.4–23.3
Tree age, years, mean (±s.e.)	93.9 ± 7.4	99.1 ± 9.9
Tree age, years, range	37–140	54–142
BAI, cm^2^ year^−1^, mean (±s.e.)^a^	3.16 ± 0.23	2.52 ± 0.15
BAI range, cm^2^ year^−1^^a^	1.47–4.39	1.67–3.91
∆^13^C, ‰, mean (±s.e.)^b^	17.51 ± 0.13	18.15 ± 0.16
∆^13^C, ‰, range^b^	16.92–18.26	17.45–19.04

^a^From annual BAI series in the period 1942–2001.

^b^From ∆^13^C quantified in 5-year intervals in the period 1903–2001.

Tree age was estimated based on the length of the longest tree-ring series of each tree (in years). Annual basal area increments (BAI; in centimetres squared per year) were calculated from raw tree-ring widths (Biondi and Qeadan, 2008), and mean BAI series were calculated at individual and sex levels. To calculate the relationships between tree-ring growth and climate for male and female trees, we standardized the BAI series by using a linear function to remove ageing-related trends in the BAI time series. To obtain a chronology of growth indices for each sex, the arithmetic mean of the standardized series of BAI indices was calculated on a year-by-year basis separately for male and female trees. A linear function was chosen for BAI standardization because most BAI series showed a linear trend. To standardize BAI series, we used the *detrendeR* software (Campelo *et al.*, 2012) in the R environment (R Core Team, 2022).

### Carbon isotopic discrimination

After cross-dating and tree-ring growth analyses, the cores were cut into groups of five-ring sequences (pentads), and these pentads were ground into a homogenized fine powder (to pass through a 40-mesh sieve) to determine their carbon isotope composition (δ^13^C). Given that the different wood components can contain different isotope fractions (Benner *et al.*, 1987) and to avoid the effects of translocation across ring boundaries of some mobile wood compounds (Tans *et al.*, 1978), isotope analyses were carried out on the holocellulose, which is a relatively immobile compound that remains limited to the rings in which it was formed. However, some authors have found similar relationships when using either whole wood or cellulose (Korol *et al.*, 1999; Warren *et al.*, 2001). Holocellulose was extracted as follows. After removal of waxes, oils and resins, a set of samples were boiled for 12 h in a Soxhlet extractor filled with a 2:l toluene–ethanol mixture. After cooling and drying for 1–2 h, the wood samples were boiled for another 12 h in the Soxhlet filled with 100 % ethanol. The samples were dried, then transferred to a beaker and boiled in deionized water for 6 h. At this stage, wood was bleached at 70 °C in acetic acid solution with the addition of glacial acetic acid and sodium hypochlorite to decompose the lignin (Leavitt and Danzer, 1993). Finally, holocellulose was obtained by immersing the samples, initially in sodium hydroxide, then in 10 % acetic acid for 10 min (Da Silveira and Sternberg, 1989).

Carbon isotope composition determination was performed on 1–2 mg holocellulose samples taken from each of the pentads using an isotope ratio mass spectrometer (MAT253, ThermoFinnigan, Bremen, Germany). The instrumental error (twice the standard deviation) associated with each observation was 0.01 ‰. The sample preparation and analysis error between repeated analyses of the same milled material was <0.22 ‰. Carbon isotope ratios (δ ^13^C; per mille), expressed relative to the composition of a standard (Pee Dee Belemnite calcium carbonate), were calculated as (δ^13^C = [(*R*_sam_/*R*_std_) − ] × 1000, where *R* refers to the ^13^C/^12^C ratio in the wood sample (*R*_sam_) and standard (*R*_std_), respectively. According to the classical two-step discrimination model (Farquhar *et al.*, 1982), the discrimination (Δ) against ^13^C relative to air was calculated as Δ^13^C = (δ^13^C_air_ − δ^13^C_wood_)/(1 + δ^13^C_wood_). Values of δ^13^C_air_ for each of the 5-year periods used for Δ^13^C determination were derived from the work of Ferrio *et al.* (2003) and the CUINSTAAR/NOAA-CMDL database (https://gml.noaa.gov/ccgg/about/global_means.html).

### Climate data

To calculate the relationships between tree-ring growth and climate, we used gridded datasets for monthly mean air temperature (Tmed), total precipitation (Prec), cloudiness (Cld) and potential evapotranspiration (PET) obtained from the Climate Explorer of the World Meteorological Organization (http://climexp.knmi.nl/; CRU TS 4.06), Climate Research Unit, University of East Anglia (Harris *et al.*, 2020), for the 0.5° latitude × 0.5° longitude area where the study site is located, and obtained monthly standardized precipitation–evaporation index (SPEI) values. The SPEI represents a standardized measure of water balance and the accumulation of water deficits/surpluses at different time scales, with negative and positive SPEI values indicating dry and wet conditions, respectively (Vicente-Serrano *et al.*, 2010). It was computed using the *SPEI* R library (Beguería and Vicente-Serrano, 2017; R Core Team, 2022).

### Statistical analyses

**Fig. 2.**
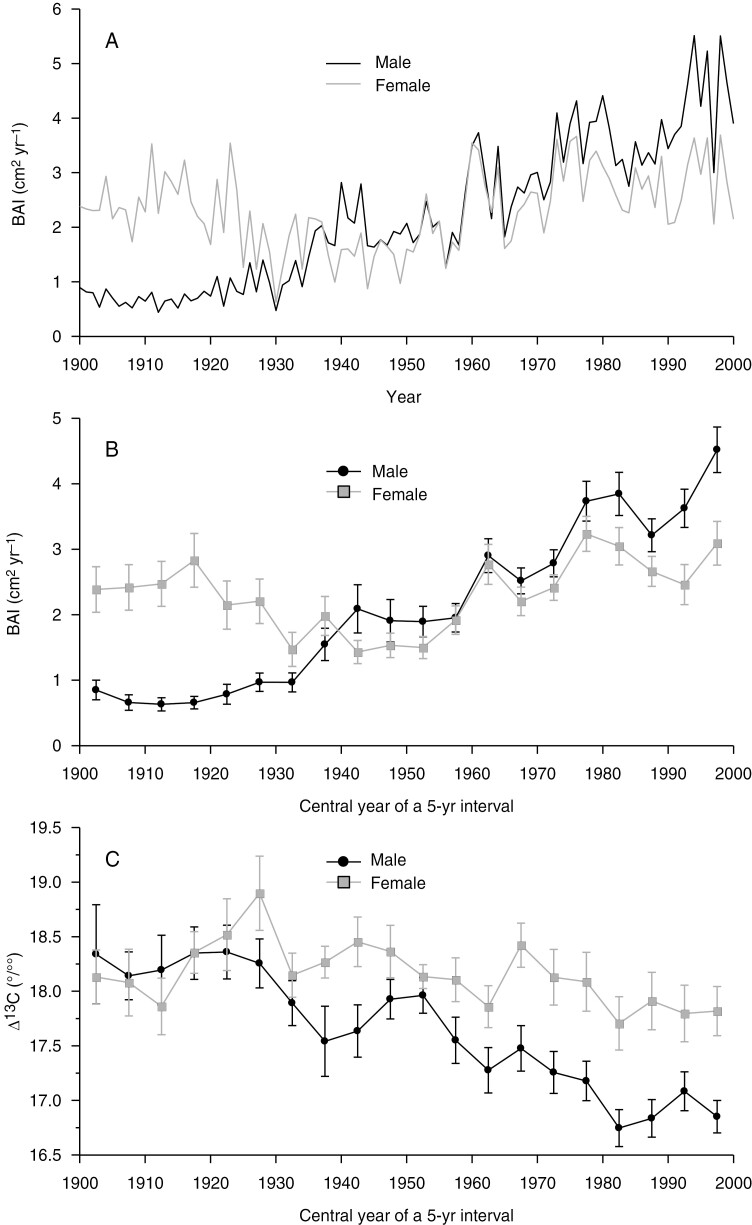
(A) Annual variation in basal area increment (BAI; in centimetres squared per year) for *Ilex aquifolium* males and females. (B, C) Variation at 5-year intervals of mean (±s.e.) BAI (B) and mean (±s.e.) carbon isotope discrimination (∆^13^C; per mille) (C) for males and females.

To compare annual growth rates and carbon isotope discrimination between the sexes, the annual BAI time series were averaged into the same five-ring sequences (pentads) in which Δ^13^C determinations were performed throughout the 20th century (Fig. 2). The relationships between BAI and Δ^13^C were calculated using linear regression, separately for male and female trees, in the considered pentads over the period 1900–2010 (*n* = 20).

We fitted linear models to find BAI and Δ^13^C differences attributable to historical tree size, ecological memory, and the interaction between time and sex. Historical tree size was reconstructed starting from the measured diameter and subtracting twice the mean individual ring width of the previous year. The ecological memory variable was defined as the previous pentad BAI and Δ^13^C value (*p−1*) matching a given pentad (*p*). The parametrization of historical tree size and ecological memory serves to avoid the possible non-independence of values obtained in repeated measures over time. We used the Akaike information criterion corrected for small sample size (AICc; Akaike, 1973) first to test whether random structures (tree identity random intercept) are required and second to performed model selection later using the *dredge* function of *MuMIn* R library (Barton, 2022). A model was considered superior if its AICc value was two or more units smaller than others (Burnham and Anderson, 2002). The best model was defined as the model with the lowest AICc and was validated by comparing it with a null model set with constant fixed effects. Both growth and isotopic discrimination models did not support the inclusion of random structures, and standard linear models were fitted instead. Finally, dependent variables were logarithmically transformed when necessary to meet the normality assumptions of linear model residuals.

The effects of monthly climatic factors on standardized BAI chronologies for male and female *I. aquifolium* trees were examined over the period 1942–2001 (*n* = 60), when the chronologies of both sexes were well replicated, by calculating their Pearson correlations with monthly time series of Tmed, Prec, Cld and PET from April of the previous year [Apr(−1)] to November of the current growth year (Nov). To test for differential legacy effects of water availability on the growth of males and females, we examined the lagged effect of water availability at different time scales during the growing year and the 2 years before growth. To this end, we calculated Pearson’s correlations between male and female standardized BAI chronologies and the SPEI from previous September (SEP) to December (Dec) of the growing year, with a lag of 1–36 months.

Finally, the significant relationships obtained from these previous correlation analyses were considered to select meaningful climate predictors for the growth of male and female *I. aquifolium* trees in the study area. To select the climatic variables most relevant to male and female growth and to avoid collinearity between them, multiple linear regression models were calculated stepwise, using standardized BAI chronologies of male and female trees as dependent variables, and the significant climate predictors between 1942 and 2001 were considered independent predictors. A stepwise multiple linear regression analysis was performed using the *olsrr* software in the R environment (R Core Team, 2022), which performs collinearity diagnosis and selects the best regression model using a stepwise procedure by entering and removing predictors based on *P*-values (Hebbali, 2020).

## RESULTS

### Tree traits and long-term BAI variation

**Fig. 3.**
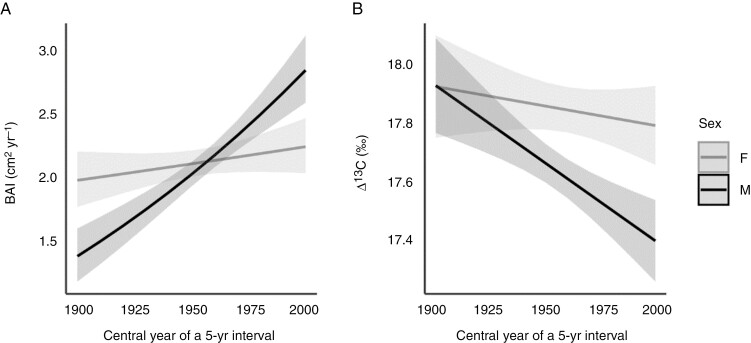
Adjusted linear predictions of the year × sex interaction term from basal area increment basal area increment (BAI) (A) and carbon isotope discrimination (∆^13^C) (B) best models (Table 2). The shaded area shows 95 % confidence intervals.

The size of the male and female *I. aquifolium* trees examined was almost identical, with a mean stem diameter of 18.5 cm and a range of 13–23 cm (Table 1). The mean age of females (99 years) was slightly older than that of males (94 years). Overall, the mean BAI of males (3.16 cm^2^ year^−1^) was greater than that of females (2.52 cm^2^ year^−1^), whereas the mean Δ^13^C value was higher in females (18.16 ‰) than in males (17.51 ‰). However, when examining the temporal trends of the mean annual BAI values (Fig. 2A) and mean BAI values in tree-ring pentads (Fig. 2B) we observed that females had higher BAI values than males from 1900 to the 1930s, that both sexes had similar BAI values from the 1940s to the 1970s, and that males have had higher BAI values since the 1980s. Indeed, these observed trends are supported by significant differences between the sexes that depended on time, as indicated by the best BAI model that supported the effects of sex, ecological memory represented by the BAI in the previous pentad, historical tree size (diameter at breast height) and the pentad × sex interaction (Table 2). The fitted linear predictions of this interaction term from BAI showed that females grew more than males during the period 1900–1930 and that males grew more than females during the period 1980–2000 (Fig. 3A).

**Table 2.** Summary of the best linear models for secondary growth (BAI) and carbon isotope discrimination (∆^13^C) during the 1900–2000 period, quantifying the individual response of *Ilex aquifolium* and showing the effects of time (pentad), sex, ecological memory (BAI_*p*−1_ and ∆^13^C_*p*−1_) and size (diameter at breast height) as covariates. The model type III *F*-statistic is shown with the associated *P*-value. Significant effects (*P* < 0.05) are highlighted in bold. Best models enhanced their null model counterpart in ∆AICc _null–best_ = 528.8 (BAI) and 463.0 (∆^13^C) units.

Best model	Model *r*_adj_^2^	Model *F*	Model *P*-value	Predictor variable	*β*	Confidence interval	*P*-value
BAI	0.682	200.5	<0.001	Pentad	0.00	−0.00 to 0.00	0.177
				Sex [male]	−1.00	−1.00 to −0.99	**<0.001**
				BAI_*p*−1_	0.20	0.18–0.23	**<0.001**
				Diameter at breast height	0.01	0.00–0.02	**0.010**
				Pentad × sex [male]	0.00	0.00–0.01	**<0.001**
Δ^13^C	0.752	254.2	<0.001	Pentad	−0.00	−0.00 to 0.00	0.330
				Sex [male]	7.59	0.31–14.88	**0.041**
				Δ^13^C_*p*−1_	0.76	0.69–0.83	**<0.001**
				Pentad × sex [male]	−0.00	−0.01 to −0.00	**0.036**

### Long-term Δ^13^C variation and its relationships with BAI

∆^13^C values in tree rings of males and females at 5-year intervals varied over time, showing similar Δ^13^C values between the sexes between 1900 and the 1930s, but maintaining higher Δ^13^C levels in females than males since the 1930s (Fig. 2C). The best-fitting model for Δ^13^C supported the effects of the pentad × sex interaction and ecological memory represented by the Δ^13^C in the previous pentad on individual Δ^13^C (Table 2). The effect of historical tree size was not supported. The fitted linear predictions of the pentad × sex interaction for Δ^13^C showed a sex-dependent difference over time, because values were higher in females than males from the 1940s to the 2000 (Fig. 3B), suggesting that females had lower water-use efficiency than males during the 1950–2000 period. The best BAI linear model accounted for the relevant effects of pentad × sex, the ecological memory of the previous 5 years and historical tree size, with the effect of the two latter terms being positive (Table 2). The slope of the time term was larger in the case of males, but female trees outperformed males during the first three decades of the century, whereas males grew more during the last two decades (Fig. 3A). Finally, sex had no effect on the temporal variation of either Δ^13^C and BAI, because the interaction term was not supported by the best models.

**Fig. 4.**
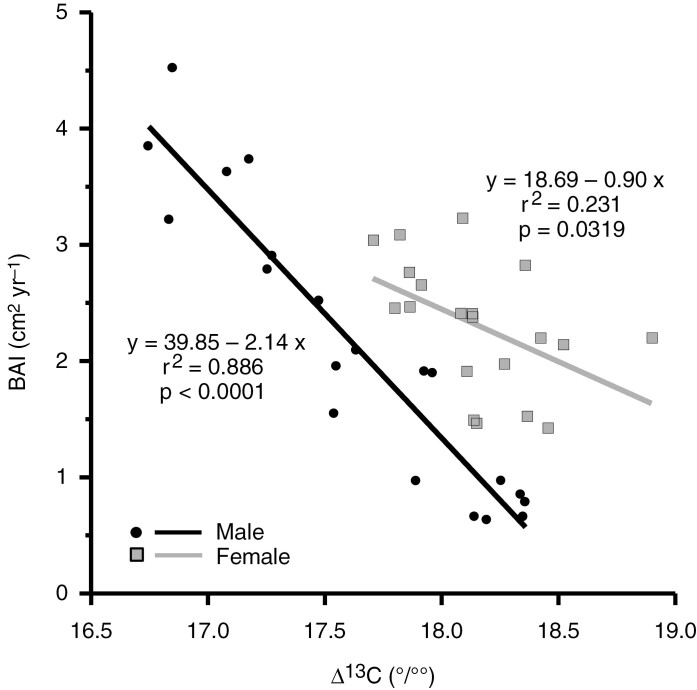
Relationships between mean basal area increment (BAI; in centimetres squared per year) and carbon isotope discrimination (∆^13^C; per mille) at 5-year intervals for males and females. The obtained linear fits, their coefficients of determination and their statistical significance are shown.

The temporal differentiation between the sexes in terms of growth rates and water-use efficiency was also evidenced by the linear relationships between BAI and Δ^13^C (Fig. 4), which were negative and significant for both sexes, but was stronger in males (*r*^2^ = 0.886, *P* < 0.0001) than in females (*r*^2^ = 0.231, *P* = 0.0319).

### Responses of radial growth to climatic variation

**Fig. 5.**
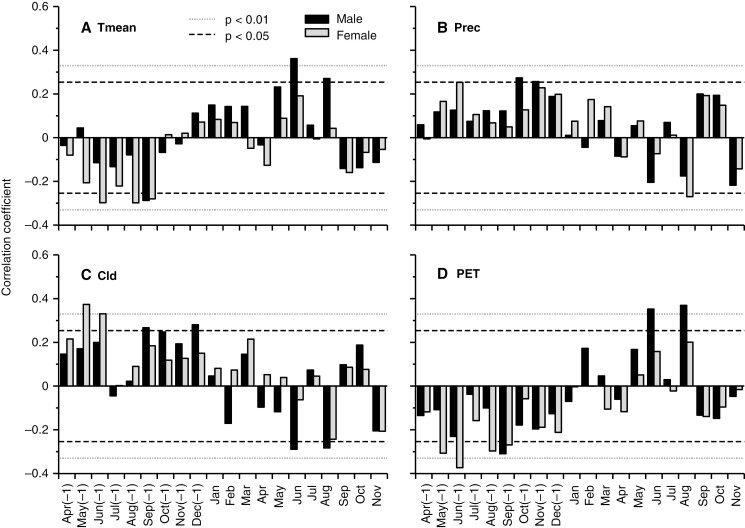
Correlations of annual standardized basal area increments (BAI) with the monthly climatic variables: (A) mean temperature (Tmean); (B) precipitation (Prec); (C) cloud cover (Cld); and (D) potential evapotranspiration (PET) for males (black bars) and females (grey bars) over the period 1942–2001. Dashed lines indicate *P* < 0.05 and dotted lines *P* < 0.01. Jan to Dec refers to the months of the year, from January to December; (−1) indicates the year before the trees grew.

The annual standardized BAI chronologies for males and females showed different correlations with the considered monthly climate time series, with some agreement between the sexes (Fig. 5). The standardized BAI of males had a significant negative correlation with mean air temperature in the previous September and positive correlations in current June and August, while females had significant negative correlations with mean air temperature only in the previous June, August and September (Fig. 5A). Regarding total monthly precipitation, standardized BAI of males had a significant negative correlation with current August precipitation, while BAI of females had significant positive correlations with precipitation in October and November of the previous year (Fig. 5B). When considering cloud cover, the BAI of males was positively correlated with cloudiness in September and December of the previous year and negatively in the current June and August, while the BAI of females was positively correlated with cloudiness in May and June of the previous year (Fig. 5C). The relationships between standardized BAI and potential evapotranspiration were found to be almost opposite to those observed for cloud cover, with a negative correlation for males in the previous September and positive correlations in the current June and August, and only negative correlations for females in May and June of the previous year (Fig. 5D).

**Fig. 6.**
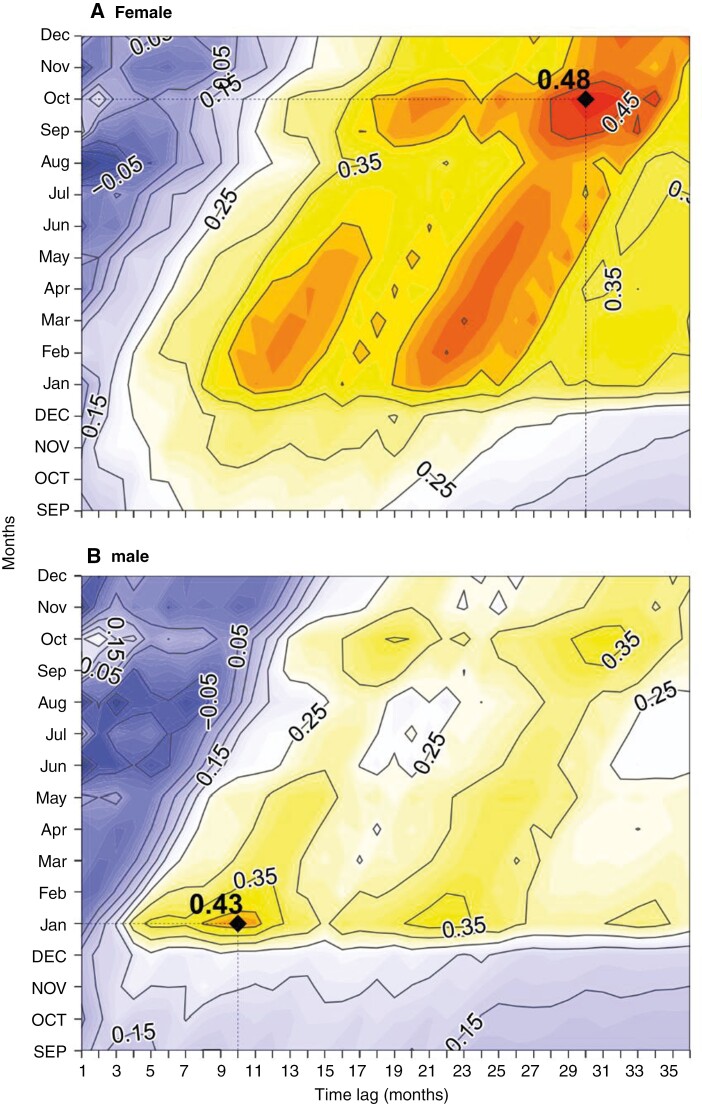
Correlation (*r*) of the standardized BAI chronologies of female (A) and male (B) *Ilex aquifolium* trees in Sierra de Ancares explained by monthly standardized precipitation–evaporation index (SPEI) series at different time scales for the period 1942–2001. The analyses were calculated from September of the previous year to December of the current growth year (previous year months are depicted in capitals). Insets in the graphs (dotted lines and black diamonds) represent the month and time lag at which the maximum correlation occurs.

A relevant delayed effect of SPEI on the standardized BAI was found for both sexes, but with different timing and magnitude. Positive correlations of >0.4 were found for lags between 9 and 36 months in females, with the highest correlation of 0.48 occurring in October, 30 months before tree growth (Fig. 6A). In males, the positive lagged effect of SPEI on standardized BAI was evidenced by correlations of >0.4 between 8 and 11 months before tree growth, with the highest correlation of 0.43 occurring in January, with a lag of 10 months (Fig. 6B).

Multiple regression models that considered the overall effect of significant climate predictors together demonstrated high statistical significance for both sexes, with climate explaining 30.4 % of the standardized BAI variation in male trees and 37.6 % in female trees (Table 3). Lagged water availability was the most important climatic predictor of BAI, with SPEI in January with a lag of 10 months being the main predictor in males and SPEI in October with a lag of 30 months in females. The mean temperature in June and potential evapotranspiration in August also showed a positive association with male BAI (Table 3). For females, cloud cover in May and June of the year before growth and the mean air temperature in August and September of the year before growth were also significant predictors of BAI (Table 3).

**Table 3.** Statistics of the stepwise multiple linear regression models for standardized basal area increment (BAI) chronologies of male and female *Ilex aquifolium* trees with the significant climatic predictors over the period 1942–2001 (*n* = 60).

Sex	Model *r*_adj_^2^	Model *F*	Model *P*-value	Predictor variable^a^	*β*	*t*-value	*P*-value
Male	0.304	9.58	<0.001	SPEI11 Jan	0.375	3.43	0.001
				Tmed Jun	0.272	2.35	0.022
				PET Aug	0.240	2.06	0.044
Female	0.376	12.83	<0.001	SPEI30 Oct	0.340	3.18	0.002
				Cld MayJun(−1)	0.325	3.06	0.003
				Tmed AugSep(−1)	–0.309	–2.78	0.004

^a^Code interpretation for climate predictors: Cld, cloudiness; PET, potential evapotranspiration; SPEI, standardized precipitation evapotranspiration index; Tmed, mean temperature. Jan to Dec means the months of the year, from January to December; (−1), year before tree growth. Two months indicate that the average of two adjacent months has been calculated. SPEI11 and SPEI30 refer to the accumulated SPEI of the last 11 and 30 months, respectively.

## DISCUSSION

### Intersex differences in growth rates, Δ^13^C and their relationships

Males and females of *I. aquifolium* differed in their secondary growth rates, quantified as BAI, with males growing more than females in recent decades. This result contradicts some studies that do not demonstrate different growth rates between the sexes of dioecious woody plants (Martins *et al.*, 2021; Garcia-Barreda *et al.*, 2022). However, our results support other studies that showed higher growth rates in males than in females (Montesinos *et al.*, 2006; Nuñez *et al.*, 2008; Cedro and Iszkuło, 2011), although the opposite pattern was also found (Rozas *et al.*, 2009; Cattaneo *et al.*, 2013; Desoto *et al.*, 2016). This is not unexpected, because sexual differentiation in growth rates can be site dependent, with males generally growing more than females in colder and higher-elevation locations (Huang *et al.*, 2018; Rozas *et al.*, 2019), and with females often growing at a higher rate in high-moisture environments (Ward *et al.*, 2002). In northern Spain, the distribution of *I. aquifolium* extends from sea level to an elevation of 1600 m, and in the Mediterranean mountains occasionally ≤1850 m (Benedí, 1997). This means that our studied population at 1400 m is in the upper elevation range of the species. Therefore, it is plausible to hypothesize that males of *I. aquifolium* grow faster than females at our study site given its high elevation, whereas trees of this species at lower elevations might even show a reversed growth differentiation between the sexes.

The differences in growth rates between the sexes of *I. aquifolium* also changed over time, with females having a higher BAI than males during the period 1900–1930, whereas from 1980 onwards the trend was reversed and males had a higher BAI than females. Temporal changes in the differences between males and females in their growth patterns have been found in other dioecious plant species (e.g. Rovere *et al.*, 2003) and have been attributed to differences in the timing and cost of reproduction between the sexes, which are expressed differently at different stages of the life cycle (Iszkuło and Boratyński, 2011; Sánchez Vilas and Pannell, 2011). Furthermore, in a previous study on 3-year-old seedlings, a greater relative growth rate was found in females than in males of *I. aquifolium* (Retuerto *et al.*, 2000). The temporal pattern observed here could be explained by the fact that males of *I. aquifolium* mature earlier and therefore invest more in reproduction (and less in growth) at a younger age than immature females, whose maturation is delayed in comparison to males (Iszkuło and Boratyński, 2011). Males tend to flower earlier in the season than females (Forrest, 2014), and although there is less empirical evidence on the life-history transition to maturity, males of some dioecious species have also been found to become reproductively viable at an earlier age than conspecific females (Purrington and Schmitt, 1998; Bram and Quinn, 2000; Meagher and Delph, 2001; Sánchez Vilas and Pannell, 2011). Although no data are available for *I. aquifolium*, males of *Ilex opaca* mature earlier than females (Clark and Orton, 1967). The earlier age at first reproduction by males has been justified by the fact that females require a longer period of resource accumulation before flowering begins in order to achieve greater reproductive effort owing to fruit production (Lloyd and Webb, 1977; Purrington and Schmitt, 1998; Meagher and Delph, 2001). However, adult females of *I. aquifolium* later invest more in reproduction than males (Obeso and Retuerto, 2002), which could limit resource allocation for growth and therefore explain why males grow more than females after maturity, as shown in other studies (Obeso *et al.*, 1998; Obeso and Retuerto, 2002). Furthermore, the increasing trend in mean temperatures in the study area over the course of the 20th century was particularly pronounced in recent decades (see Fig. 1C). Given that the growth of females is more affected by increased temperatures than that of males, this increasing temperature would have a greater impact on females than on males.

Carbon isotope discrimination (Δ^13^C) of male and female tree rings did not differ from 1900 to 1930, but from 1930 onwards females had higher Δ^13^C levels, perhaps reflecting a less conservative water use than males. Females are expected to use water less conservatively than males, as has been noted previously (Rozas *et al.*, 2009; Tognetti, 2012; Hultine *et al.*, 2013), probably because females have higher resource requirements than males owing to fruit production (Obeso, 2002; Harris and Pannell, 2010; but see Roddy *et al.*, 2019; Midgley and Cramer, 2022). Similar results have been found in other dioecious species, such as *Acer negundo* (Dawson and Ehleringer, 1993) or *Corema album* (Díaz-Barradas *et al.*, 2018). However, there are also studies that found no differences in carbon isotope discrimination between the sexes (Garcia-Barreda *et al.*, 2022) or if they did exist, they were context dependent (Retuerto *et al.*, 2000). Considering that females of *I. aquifolium* are less conservative in the use of water than males, this could make them more vulnerable to drought than males, especially in drier environments (Dawson and Ehleringer, 1993; Retuerto *et al.*, 2000; Hultine *et al.*, 2013). These findings could therefore be important in predicting the potential response of this species to global change. In particular, an increase in drought and/or temperature is expected across the entire range of *I. aquifolium* (IPCC, 2023), which could impact the sex ratio of the populations. The sex ratio would become male biased, hence the risk of population extinction would increase owing to a local lack of females (Cruden, 2000; Iszkuło *et al.*, 2009; Tognetti, 2012; Petry *et al.*, 2016).

We found a negative relationship between BAI and Δ^13^C, suggesting that higher water-use efficiency resulted in greater radial growth. Notably, the relationship between BAI and Δ^13^C was stronger in males, indicating a greater physiological connection between growth and carbon isotope discrimination in males than in females of *I. aquifolium*. For other tree species, such as the angiosperms *Populus euphratica* (Liu *et al.*, 2014) and *Populus orientalis* (Weiwei *et al.*, 2018) and the gymnosperms *Juniperus thurifera* (Granda *et al.*, 2014) and *Pinus densiflora* (Kwak *et al.*, 2016), long-term increases in BAI concomitant with increases in water-use efficiency were found. However, this pattern is not always present (Nock *et al.*, 2011; Wu *et al.*, 2015), suggesting that for some tree species, or for a particular species growing in different environmental conditions (Brienen *et al.*, 2022; Urrutia-Jalabert *et al.*, 2022), it is more difficult to overcome the negative effects of severe constraints by increasing water-use efficiency (Heilman *et al.*, 2021). A better understanding of the long-term relationship between growth rates and water-use efficiency is crucial to improving our ability to predict how plants might respond to future climate changes (Weiwei *et al.*, 2018; Heilman *et al.*, 2021), particularly for dioecious plant species.

### Influence of sex on the climatic response of secondary growth

Climatic variables significantly influenced the annual growth rates of *I. aquifolium*, with males and females showing different climate sensitivity. Sex-related sensitivity of tree-ring growth to climate has already been reported in a variety of dioecious woody species (Montesinos *et al.*, 2006; Rozas *et al.*, 2009; Iszkuło and Boratyński, 2011; Cattaneo *et al.*, 2013; Huang *et al.*, 2018). In some species, females have been found to be more sensitive to certain climatic variables, such as precipitation (Iszkuło *et al.*, 2009; Rozas *et al.*, 2019), but the patterns appear to be complex, given that sex-specific sensitivity to climate has been found to depend on the environmental context, such as local variations in radiation exposure or elevation (Nuñez *et al.*, 2008; Huang *et al.*, 2018).

Overall, females were more sensitive to the climatic factors of the previous year than males, whereas males were more influenced by the climatic factors of the current growing year (Fig. 5). Female growth had a positive association with cloud cover but a negative association with mean air temperature and potential evapotranspiration in the previous spring and summer, suggesting that summer water stress would significantly reduce female tree growth in the next growing season. Temperature and cloud cover are negatively correlated (Mendoza *et al.*, 2021), and opposite effects on tree-ring growth would be expected. Strong negative associations of female growth with past summer temperature have previously been found in several tree species, such as *T. baccata* (Cedro and Iszkuło, 2011) and *Araucaria araucana* (Rozas *et al.*, 2019). This is likely to be because warm summers trigger a masting event the following year, which, in turn, reduces female ring width owing to the trade-off between growth and reproduction, resulting in a negative correlation of growth with temperature of the previous summer (Hacket-Pain *et al.*, 2015). In contrast, cloudy and rainy conditions last autumn and winter promoted the growth of male trees, for which there was no previous evidence in the literature. More importantly, male growth was positively related to mean air temperature and evapotranspiration in June and August and negatively related to cloud cover in June and August. These results suggest that thermal and lighting conditions that promote high rates of transpiration and photosynthesis in summer are important for the annual growth of male *I. aquifolium* trees. Concurrently, it has been shown that the growth of male *Austrocedrus chilensis* trees is more favoured by high radiation levels than the growth of female trees (Nuñez *et al.*, 2008). The importance of summer conditions on male growth could be related to the fact that flowering in *I. aquifolium* occurs in May–June, and a greater investment in flower production would reduce the available resources that male trees could invest in growth. The differences between sexes in environmental sensitivity found here are complex, but as suggested above these might be related to their differential timing in sink strength as a function of different reproductive phenologies (Iszkuło and Boratyński, 2011).

Lagged correlations between radial growth of *I. aquifolium* and water availability, estimated by cumulative SPEI during the last 36 months, showed that males had a greater sensitivity to drought in a 10-month period, whereas females showed greater sensitivity to drought in a 30-month period. These extremely delayed drought legacies can be observed in trees growing in arid or semi-arid environments under chronic water deficit (Peltier and Ogle, 2019; Rozas *et al.*, 2021). However, our results revealed that female *I. aquifolium* trees growing in a temperate climate are also more influenced by past climatic conditions than males, suggesting that female trees have greater drought memory than male trees (Peltier and Ogle, 2023). The SPEI sensitivity was greater in females than in males (0.48 in females vs. 0.43 in males; Fig. 6), although female growth was also highly correlated with SPEI ranging from February to June, with a lag of 21–26 months, and during February–March, with a lag of 12–13 months. This was probably because resource budgets and weather are involved in the functional mechanisms that drive vegetative growth and reproduction, and both processes would then be coupled in the long term, particularly for females (Montesinos *et al.*, 2006; Pearse *et al.*, 2016). Interestingly, Garcia-Barreda *et al.* (2022) showed that the lagged sex-specific effects of drought stress on growth can be also species specific, with the medium-term effect of water availability being larger in males in some species but larger in females in others.

Considering that trees of both sexes undergo the same environmental fluctuations, differences might be owing to functional traits related to reproduction because, as pointed above, adult female trees of this species usually incur higher costs of reproduction than adult male trees (Obeso, 1997; Obeso and Retuerto, 2002). Different climatic affinities resulting from sexual differentiation would ultimately indicate a compensatory functional response of growth (DeClerk *et al.*, 2006; Tornos-Estupiña *et al.*, 2023). However, for populations growing at the rear- or dry-edge of the distribution range, the disadvantages might easily outweigh the advantages, because the trend of rising temperatures is constraining tree growth (Shestakova *et al.*, 2016). High dependence on climate poses a high risk of growth changes during and in the years immediately after drought episodes (Peltier and Ogle, 2020), which can lead to tree malfunction and tree death (Adams *et al.*, 2017). In general, males exhibit more efficient water use and are generally less sensitive to increased drought than co-occurring females (Hultine *et al.*, 2016, 2018); therefore, a male-biased sex ratio is possible in the future in a significant number of populations.

## Conclusions

We found that sex differences in secondary growth rates were unstable over time, with males outperforming females in both radial growth rates and water-use efficiency in recent decades. Additionally, males showed a stronger relationship between water-use efficiency and growth rates than females, which is likely to be attributable to the higher reproductive effort of females compared with males. Furthermore, our results showed a sex-dependent response of secondary growth to climatic conditions, with summer water stress significantly reducing the growth of female trees in the next growing season. In contrast, male tree growth was favoured primarily by cloudy and rainy conditions last autumn and winter, combined with low cloud cover and warm conditions in summer. This study is the first to report sex-dependent lagged correlations between *I. aquifolium* radial growth and water availability, with the strongest relationship between tree growth and cumulative water availability at 30 months in females and at 10 months in males. Therefore, we suggest that male growth depends on water availability on short time scales, whereas female growth is related to cumulative water availability over medium to long time scales. It is likely that these differences between the sexes arise because the functional mechanisms that drive vegetative growth and reproduction depend on past weather, which determines current resource budgets, and both processes are more strongly coupled in the long term in females than in males.

## Supplementary Material

mcae126_suppl_Supplementary_Material

## Data Availability

The data that support the findings of this study are available from the authors upon reasonable request.
