# Correction to: Differential growth rate, water-use efficiency and climate sensitivity between males and females of *Ilex aquifolium* in north-western Spain

**DOI:** 10.1093/aob/mcae175

**Published:** 2024-10-16

This is a correction to: Julia Sánchez Vilas, Héctor Hernández-Alonso, Vicente Rozas, Rubén Retuerto, Differential growth rate, water-use efficiency and climate sensitivity between males and females of *Ilex aquifolium* in north-western Spain, *Annals of Botany*, Volume 135, Issue 1–2, 1 January 2025, Pages 357–370, https://doi.org/10.1093/aob/mcae126

In the originally published version of this paper, the Y axis of Figure 1C was labelled as “Mean temperature (mm)”. This has been corrected to “Mean temperature (°C).

