# Supplementary figures and images for: Differential growth rate, water-use efficiency and climate sensitivity between males and females of Ilex aquifolium in north-western Spain
Source: Ann Bot. 2024 Aug 7;135(1-2):357–70. doi: 10.1093/aob/mcae126 (PMC11805936; doi:10.1093/aob/mcae126)

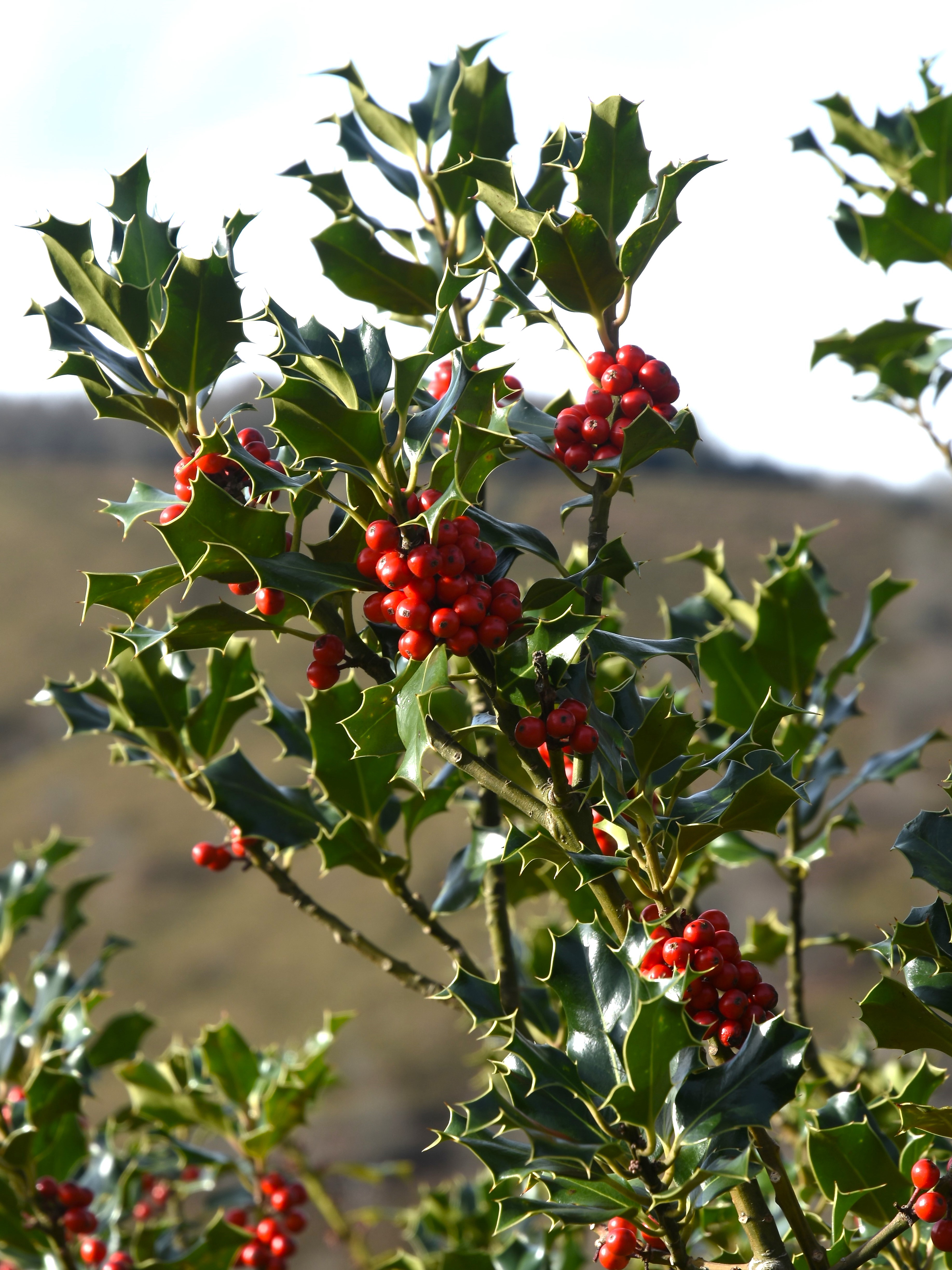

Supplement: mcae126_suppl_Supplementary_Material [file mcae126_suppl_supplementary_material.zip › aob-24204-s03.jpg]
