# Supplementary material for: Differential growth rate, water-use efficiency and climate sensitivity between males and females of Ilex aquifolium in north-western Spain
Source: Ann Bot. 2024 Aug 7;135(1-2):357–70. doi: 10.1093/aob/mcae126 (PMC11805936; doi:10.1093/aob/mcae126)
Supplement: mcae126_suppl_Supplementary_Material [file mcae126_suppl_supplementary_material.zip › aob-24204-s02.doc]

Differential growth rate, water use efficiency and climate sensitivity between males and females of *Ilex aquifolium* in north-western Spain

^1,2,*^Sánchez Vilas, Julia, ^3,4^Hernández-Alonso, Héctor, ^3^Rozas, Vicente & ^1^Retuerto, Rubén

^1^Departamento de Bioloxía Funcional (Área de Ecoloxía), Facultade de Bioloxía, Universidade de Santiago de Compostela, c/ Lope Gómez de Marzoa s/n, 15782 Santiago de Compostela, Spain

^2^School of Biosciences, Sir Martin Evans Building, Cardiff University, CF10 3AX Cardiff, UK

^3^EiFAB, iuFOR, Universidad de Valladolid, Campus Duques de Soria, 42004 Soria, Spain

^4^CEFE, Univ Montpellier, CNRS, EPHE, IRD, Montpellier, France

***author for correspondence**

e-mail: sanchezvilasj@cardiff.ac.uk

**Short title**: Intersexual differences in growth, WUE and climate sensitivity in *I. aquifolium*

**Keywords**: dendroecology, dioecy, sexual dimorphism, tree growth, carbon isotope discrimination, water use efficiency

**Highlight:**

Males and females of *I. aquifolium* have different patterns of growth and different sensitivity to climatic variables, pointing to greater vulnerability of females to increasing drought, which could lead to sex-ratio biases threatening population viability in the future.

Figures: 6

Tables: 3

**Abstract**

**Background and aims** Dioecious plant species, i.e., those in which male and female functions are housed in different individuals, are particularly vulnerable to global environmental changes. For long-lived plant species, such as trees, long-term studies are imperative to understand how growth patterns and their sensitivity to climate variability differentially affect the sexes.

**Methods** Here, we explore long-term intersexual differences in wood traits, namely radial growth rates, water use efficiency quantified as stable carbon isotope abundance of wood cellulose, and their climate sensitivity in *Ilex aquifolium* trees growing in a natural population in NW Spain.

**Key results** We found that sex differences in secondary growth rates were variable over time, with males outperforming females in both radial growth rates and water use efficiency in recent decades. Summer water stress significantly reduced the growth of female trees in the following growing season, while the growth of male trees was primarily favoured by cloudy and rainy conditions the previous fall and winter combined with low cloud cover and warm conditions in summer. Sex-dependent lagged correlations between radial growth and water availability were found, with a strong association between tree growth and cumulative water availability in females at 30 months and in males at 10 months.

**Conclusions** Overall, our results point to greater vulnerability of female tress to increasing drought, which could lead to sex-ratio biases threatening population viability in the future.

**Introduction**

Dioecy, in which separate male and female individuals coexist in a population, is a relatively rare sexual system in flowering plants occurring in approximately 6% of species (Renner 2014). Nevertheless, dioecy has evolved independently many times and is widespread in angiosperms, occurring in almost half of all angiosperm families (Heilbuth 2000; Vamosi et al. 2003; Renner 2014). Avoidance of self-fertilization and the corresponding expression of inbreeding depression, and sexual specialization have been claimed as two broad reasons for the selection of separate sexes (see review article (Pannell and Jordan 2022)). Once dioecy occurs, females and males are expected to differentiate and specialize according to their individual sexual roles and associated resource needs, which can result in functional and performance differences between the sexes (Obeso 2002; Barrett and Hough 2013). Sexual dimorphism can occur not only in the reproductive organs, but also in the morphology, physiology, and life history traits, which is also known as secondary sexual dimorphism (Obeso 2002; Barrett and Hough 2013). Such differences have been attributed to resource trade-offs between allocation to reproduction and to other functions such as growth and maintenance (Obeso 2002).

The general assumption is that females of dioecious woody plants usually expend proportionally more resources on reproduction because they produce not only flowers but also fruits (Obeso 2002; Barrett and Hough 2013), and hence they are expected to grow less and perform worse under environmental stress (Juvany and Munné-Bosch 2015). However, there is still no universal pattern, and some studies have observed equal or even greater reproductive effort in males and greater growth and performance in females (Barrett and Hough 2013; Juvany and Munné-Bosch 2015). It was found that the presence and direction of differences between males and females are species-specific and highly context-dependent (Retuerto et al. 2000, 2018; Juvany and Munné-Bosch 2015). Therefore, there is a need for further data, particularly on sex-related differences in physiology of dioecious woody angiosperms in response to environmental stress.

The physiological aspects have been less studied than morphological or growth aspects, probably due to the association of dioecy with a set of life history traits (e.g., size, woodiness, perenniality) that challenge physiological measurement, integration and generalization (Vamosi et al. 2003). However, the physiological comparison between the sexes is of great relevance because compensatory mechanisms can play a role in mitigating reproductive costs and differences in physiological performance can lead to sexual differences in growth, survival and even population structure (Retuerto et al. 2006, 2018; Juvany and Munné-Bosch 2015). Physiological comparison between the sexes has been studied primarily using leaf-scale instantaneous measurements, such as photosynthetic activity, transpiration rate and other water-relation parameters (Dawson and Ehleringer 1993; Dawson et al. 2004). However, short-term studies or single time-point studies can be misleading because the sexes may differ in their developmental timing, including the timing of investment in reproductive allocation (Milla et al. 2006; Sánchez Vilas and Pannell 2011) and also in the frequency and intensity of reproductive events (Obeso 2002). In this context, further research is needed to thoroughly understand and realistically model the quantitative relationships between male and female reproductive efforts over the entire growing season or life cycle, particularly in woody species. This aspect is particularly important in the current context of climate change, because if growth and physiological responses to factors such as rising air temperature, increasing drought and CO_2_ concentration differ between the sexes of dioecious species, this could make them more vulnerable to changes in sex ratios and potentially leading to population decline and eventual extinction (Tognetti 2012; Petry et al. 2016).

Dendroecological studies are promising at comparing wood traits of males and females by providing a holistic, comprehensive and long-term analysis of tree responses to environmental changes. Radial growth of the woody stem is a highly integrative trait that is useful for assessing long-term plant efficiency as it reflects the overall performance of metabolic processes (Eckes-Shephard et al. 2022). In addition, stable carbon isotopes in tree rings provide time-integrated insights into ecophysiological processes related to plant water stress (Gessler et al. 2014), such as leaf conductance, hydraulic capacity and photosynthetic capacity (Körner et al. 1988; Farquhar et al. 1989; Ehleringer 1993), pre-dawn water potential (Damesin et al. 1998; Warren et al. 2001), climatic gradients of air humidity (Saurer and Siegenthaler 1989) or precipitation (Korol et al. 1999; Warren et al. 2001; Hartman and Danin 2010). Carbon isotope discrimination (Δ^13^C), which reflects the degree of discrimination against the ^13^C isotope, is often used to quantify the water-use efficiency (WUE), i.e. the ratio of net photosynthesis to stomatal conductance to water vapor, integrated over time (Farquhar et al. 1982; Seibt et al. 2008). Differences in Δ^13^C between the sexes of dioecious plants have been reported (Dawson and Bliss 1989; Dawson and Ehleringer 1993; Retuerto et al. 2000). To improve our understanding of the ecological meaning of Δ^13^C levels, Ferrio et al. (2003) have suggested supplementing Δ^13^C analyses with other indicators of plant function, such as morphological traits (Fleck et al. 1996; Damesin et al. 1997) or plant growth (Dupouey et al. 1993; Brooks et al. 1998).

The aim of this study is to examine long-term intersexual differences in wood traits, namely radial growth and stable carbon isotopes, and its climate sensitivity in the dioecious tree *Ilex aquifolium* (European holly) growing in a natural population in Galicia, north-western Spain. Specifically, we aim (1) to determine whether sexual differences in secondary growth rates are stable over time, and (2) to investigate whether there is a sex-dependent response of secondary growth to climatic conditions in male and female trees. Previous research identified significant differences in the physiological responses of male and female *I. aquifolium* trees to resource availability (Retuerto et al. 2000), but efforts to contrast these results on a wide temporal scale and to assess possible sex-dependent effects on temporal stability remain unexplored. Therefore, we expect different growth rates, water use efficiency and climate sensitivity between males and females of *I. aquifolium*.

**Material and Methods**

**Study species**

*Ilex aquifolium* (Aquifoliaceae), the European holly, is a broad-leaved evergreen tree or shrub up to 23 m tall that often grows in the understory of temperate forests, such as in our study site, the Sierra de Ancares, a protected area in the northwest of Spain (see further details below). This species was chosen due to its abundance in the study site and due to its relevant ecological role as a persistent (evergreen) understory providing important habitat and food to woodland birds and grazers (Pollo et al. 2005). The natural range of *I. aquifolium* extends from north-western Europe (64 ºN), where it is restricted to coastal areas, through central and southern Europe to North Africa (Algeria and Tunisia) and Asia Minor (34 ºN), where it grows upward from sea level into mountainous areas (Peterken and Lloyd 1967). Holly does not occur in areas where the average temperature in January is below -4.6 ºC and the average temperature of the warmest month does not exceed 12 ºC (De Candolle 1855; Iversen 1944). Depending on substrate requirements, holly grows in different soil types, from acidic podzols to chalk soils or limestone rocks (Peterken and Lloyd 1967). The flowers, which are clustered axillary on shoots that emerged the previous year, are dioecious or, very rarely, hermaphroditic (Ward 1904). Individuals can produce flowers from May to June from the tenth year, almost never if the individuals are less than 1.5 m tall. The fruit is a scarlet spherical drupe (7-12 mm) containing four pyrenes and is typically dispersed by birds. Fruit production varies greatly from year to year (Guitián and Bermejo 2006), but good fruiting years appear to correlate with the duration of sunshine in July and the air temperature of the previous year, as well as the absence of severe late-spring frosts (Peterken and Lloyd 1967). Fruits ripen in late autumn and usually last throughout the winter. It was reported that the reproductive allocation (the ratio of mass of sexual structures to branch mass) was 56 ± 87% (mean ± 1 SD) of the branch dry mass in females at the time of fruiting and 7 ± 3% in males at the time of flowering (Obeso 1997). Wood is hard and heavy, with small vessels characteristically arranged in radial rows (Schweingruber and Baas 2011).

**Study site**

The study was conducted at the site of “Cavana Vella” (Lugo, Galicia, north-western Spain), in the Sierra de Ancares, on the western edge of the Cantabrian Range. Sierra de Ancares is a protected area and was declared a UNESCO Biosphere Reserve in 2006. The studied area, located on a north-facing slope, was delimited by coordinates 6°52'43"W - 6°54'8"W (longitude) and 42°47'52"N - 42°48'2"N (latitude), with a mean altitude of 1400 m asl (Fig. 1A). The area belongs to the Eurosiberian biogeographical region but is very close to the Mediterranean region. Annual mean precipitation is 938 mm and annual mean temperature is 9.2 ºC (Fig. 1B), mean temperature in the coldest month is -2.1 ºC and the mean temperature in the warmest month is 15.7 ºC. An increasing trend in mean annual temperature was observed in the study area (Fig. 1C). The sampling site is an old-growth forest dominated by *Ilex aquifolium*, with a much lower density of other woody species (*Corylus avellana*, *Quercus petraea*, *Sorbus aucuparia*, *Betula alba* and *Taxus baccata*).

**Sampling procedure and tree-ring growth analysis**

In July 2002, 70 mature, dominant and healthy *I. aquifolium* trees, 35 of each sex, were selected for sampling in the study area. Tree bole diameter was measured, and trees were cored to the pith at breast height using a Pressler increment borer. Three 5 mm diameter wood cores were taken from each tree from southern, eastern and western exposures to facilitate cross-dating of the tree-ring series. The cores were dried for three weeks and polished with progressively finer sandpaper until the cellular structure of the xylem was clearly visible. The years corresponding to each ring were then identified and the annual ring widths (mm) were measured to the nearest 0.01 mm using a dendrochronometer (digital positiometer, Kütschenreiter, Austria) and then cross-dated. Ring-width series were cross-dated to match similar relative ring width patterns across years using distinctive ring features for different years that allowed mutual identification between trees. The quality of the cross-dating was checked using COFECHA software (Holmes 1983) and possible dating errors in the tree-ring series were identified and corrected. Due to difficulties in cross-dating, we were only able to clearly assign rings with years in complete cores from 16 male and 14 female trees (Table 1).

Tree age was estimated based on the length of the longest tree-ring series of each tree in years. Annual basal area increments (BAI, cm^2^ year^-1^) were calculated from raw tree-ring widths (Biondi and Qeadan 2008), and mean BAI series were calculated at individual and sex levels. To calculate the relationships between tree-ring growth and climate for male and female trees, we standardized the BAI series by using a linear function to remove aging-related trends in the BAI time series. To obtain a chronology of growth indices for each sex, the arithmetic mean of the standardized series of BAI indices was calculated on a year-by-year basis separately for male and female trees. A linear function was chosen for BAI standardization because most BAI series showed a linear trend. To standardize BAI series, we used the *detrendeR* software (Campelo et al. 2012) in the R environment (R Core Team 2022).

**Carbon isotopic discrimination**

After cross-dating and tree-ring growth analyses, the cores were cut into groups of five-ring sequences (pentads) and these pentads ground into a homogenized fine powder (to pass through a 40-mesh sieve) to determine their carbon isotope composition (δ^13^C). Since the different wood components can undergo different isotope fractions (Benner et al. 1987) and to avoid the effects of translocation across ring boundaries of some mobile wood compounds (Tans et al. 1978), isotope analyses were carried out on the holocellulose, which is a relatively immobile compound that remains limited to the rings in which it was formed. However, some authors have found similar relationships when using either whole wood or cellulose (Korol et al. 1999; Warren et al. 2001). Holocellulose was extracted as follows. After removal of waxes, oils and resins, a set of samples were boiled for 12 hours in a Soxhlet extractor filled with a 2:l toluene/ethanol mixture. After cooling and drying for 1-2 hours, the wood samples were boiled for another 12 hours in the Soxhlet filled with 100% ethanol. The samples were dried, then transferred to a beaker and boiled in deionized water for 6 hours. At this stage, wood was bleached at 70 ºC in acetic acid solution with the addition of glacial acetic acid and sodium hypochlorite to decompose the lignin (Leavitt and Danzer 1993). Finally, holocellulose was obtained by immersing the samples, first in sodium hydroxide and then in 10% acetic acid for 10 min (Da Silveira and Sternberg 1989).

Carbon isotope composition determination was performed on 1-2 mg holocellulose samples taken from each of the pentads using an isotope ratio mass spectrometer (MAT253, ThermoFinnigan, Bremen, Germany). The instrumental error (twice the standard deviation) associated with each observation was 0.01 ‰. The sample preparation and analysis error between repeated analyses of the same milled material was less than 0.22 ‰. Carbon isotope ratios (δ^13^C, ‰), expressed relative to the composition of a standard (Pee Dee Belemnite calcium carbonate), were calculated as (δ^13^C = [(R_sam_/R_std_)-1]×1000, where R refers to the ^13^C/^12^C ratio in the wood sample (R_sam_) and standard (R_std_), respectively. According to the classical two-step discrimination model (Farquhar et al. 1982), the discrimination (Δ) against ^13^C relative to air was calculated as Δ^13^C = (δ^13^C_air_ – δ^13^C_wood_)/(1 + δ^13^C_wood_). Values of δ^13^C_air_ for each of the five-year periods used for Δ^13^C determination were derived from Ferrio et al. (2003) and the CUINSTAAR/NOAA-CMDL database <ftp://ftp.cmdl.noaa.gov/ccg/co2c13/flask/readme.html>).

**Climate data**

To calculate the relationships between tree-ring growth and climate, we used gridded datasets for monthly mean air temperature (Tmed), total precipitation (Prec), cloudiness (Cld) and potential evapotranspiration (PET) obtained from the Climate Explorer of the World Meteorological Organization (<http://climexp.knmi.nl/>; CRU TS 4.06), Climate Research Unit, University of East Anglia (Harris et al. 2020), for the 0.5º latitude × 0.5º longitude area where the study site is located, and obtained monthly SPEI values. The SPEI represents a standardized measure of water balance and the accumulation of water deficits/surpluses at different time scales, with negative and positive SPEI values indicating dry and wet conditions, respectively (Vicente-Serrano et al. 2010). It was computed using the *SPEI* R library (Beguería and Vicente-Serrano 2017; R Core Team 2022).

**Statistical Analyses**

To compare annual growth rates and carbon isotope discrimination between the sexes, the annual BAI time-series were averaged into the same five-ring sequences (pentads) in which Δ^13^C determinations were performed throughout the 20^th^ century (Fig. 2). The relationships between BAI and Δ^13^C were calculated using linear regression, separately for male and female trees, in the considered pentads over the period 1900–2010 (n = 20).

We fitted linear models to find BAI and Δ^13^C differences attributable to historical tree size, ecological memory, and the interaction between time and sex. Historical tree size was reconstructed starting from the measured diameter and subtracting twice the mean individual ring-width of the previous year. The ecological memory variable was defined as the previous pentad BAI and Δ^13^C value (*p-1*) matching a given pentad (*p*). The parametrization of historical tree-size and ecological memory serves to avoid the possible non-independence of values obtained in repeated measures over time. We used the Akaike Information Criterion corrected for small sample size (AICc, (Akaike 1973)) to first test whether random structures (tree identity random intercept) are required and second to performed model selection later using the *dredge* function of *MuMIn* R library (Barton 2022). A model was considered superior if its AICc value was 2 or more units smaller than others (Burnham and Anderson 2002). The best model was defined as the model with the lowest AICc and was validated by comparing it with a null model set with constant fixed effects. Both growth and isotopic discrimination models did not support the inclusion of random structures and standard linear models were fitted instead. Finally, dependent variables were log-transformed when necessary to meet the normality assumptions of linear model residuals.

The effects of monthly climatic factors on standardized BAI chronologies for male and female *Ilex aquifolium* trees were examined over the period 1942–2001 (n = 60), when the chronologies of both sexes were well replicated, by calculating their Pearson correlations with monthly time series of Tmed, Prec, Cld and PET from April of the previous year (Apr(-1)) to November of the current growth year (Nov). To test for differential legacy effects of water availability on the growth of males and females, we examined the lagged effect of water availability at different time scales during the growing year and the two years before growth. To this end, we calculated Pearson’s correlations between male and female standardized BAI chronologies and the SPEI from previous September (SEP) to December (Dec) of the growing year, with a lag of 1 to 36 months.

Finally, the significant relationships obtained from these previous correlation analyses were considered to select meaningful climate predictors for the growth of male and female *I. aquifolium* trees in the study area. To select the climate variables most relevant to male and female growth and to avoid collinearity between them, multiple linear regression models were calculated stepwise, using standardized BAI chronologies of male and female trees as dependent variables, and the significant climate predictors between 1942 and 2001 were considered independent predictors. A stepwise multiple linear regression analysis was performed using the *olsrr* software in the R environment (R Core Team 2022), which performs collinearity diagnosis and selects the best regression model using a stepwise procedure by entering and removing predictors based on *p*-values (Hebbali 2020).

**Results**

**Tree traits and long-term BAI variation**

The size of the male and female *Ilex aquifolium* trees examined was almost identical, with a mean stem diameter of 18.5 cm and a range of 13–14 to 23 cm (Table 1). The mean age of females (99 years) was slightly higher than that of males (94 years). Overall, the mean BAI of males (3.16 cm^2^ yr^-1^) was greater than that of females (2.52 cm^2^ yr^-1^), whilst the mean Δ^13^C value was higher in females (18.16 ‰) than in males (17.51 ‰). However, when examining the temporal trends of the mean annual BAI values (Fig. 2A) and mean BAI values in tree-ring pentads (Fig. 2B) we observed that females had higher BAI values than males from 1900 to the 1930s, that both sexes had similar BAI values since the 1940s to the 1970s, and that males have had higher BAI values since the 1980s. Indeed, these trends are supported by significant differences between the sexes that depended on time as indicated by the best BAI model that supported the effects of sex, ecological memory represented by the BAI in the previous pentad, historical tree size (DBH), and the Pentad × Sex interaction (Table 2). The fitted linear predictions of this interaction term from BAI showed that females grew more than males during the period 1900–1930, and that males grew more than females during the period 1980–2000 (Fig. 3A).

**Long-term Δ^13^C variation and its relationships with BAI**

Differences in mean Δ^13^C values in tree rings of males and females at 5-years intervals varied over time, showing similar Δ^13^C values between the sexes between 1900 and the 1930s, but maintaining higher Δ^13^C levels in females than males since the 1930s (Fig. 2C). The best fitted model for Δ^13^C supported the effects of the Pentad × Sex interaction and ecological memory represented by the Δ^13^C in the previous pentad on individual Δ^13^C (Table 2). The effect of historical tree size was not supported. The fitted linear predictions of the Pentad × Sex interaction for Δ^13^C showed a sex-dependent difference over time, as values were higher in females than males from the 1940s to the 2000 (Fig. 3B), suggesting that females had lower water use efficiency than males during the 1950–2000 period. The best BAI linear model accounted for the relevant effects of Pentad × Sex, the ecological memory of the previous 5 years and historical tree size, with the effect of the two latter terms being positive (Table 2). The slope of the time term was larger in the case of males, but female trees outperformed males during the first 3 decades of the century while males grew more during the last 2 decades (Figure 3A). Finally, sex had no effect on the temporal variation of either Δ^13^C and BAI, as the interaction term was not supported by the best models.

The temporal differentiation between the sexes in terms of growth rates and water use efficiency was also evidenced by the linear relationships between BAI and Δ^13^C (Fig. 4), which were negative and significant for both sexes, but was stronger in males (r^2^ = 0.886, *p* < 0.0001) than in females (r^2^ = 0.231, *p* = 0.0319).

**Responses of radial growth to climate variation**

The annual standardized BAI chronologies for males and females showed different correlations to the considered monthly climate time series, with some agreement between the sexes (Fig. 5). The standardized BAI of males had a significant negative correlation with mean air temperature in the previous September and positive correlations in current June and August, while females only had significant negative correlations with mean air temperature in previous June, August and September (Fig. 5A). Regarding total monthly precipitation, standardized BAI of males had a significant negative correlation with current August precipitation, while BAI of females had significant positive correlations with the previous year’s October and November precipitation (Fig. 5B). When considering cloud cover, the BAI of males was positively correlated with cloudiness in September and December of the previous year and negatively in current June and August, while the BAI of females was positively correlated with cloudiness in May and June of the previous year (Fig. 5C). The relationships between standardized BAI and potential evapotranspiration were found to be almost opposite to those observed for cloud cover, with a negative correlation for males in the previous September and positive correlations in current June and August, and only negative correlations for females in May and June of the previous year (Fig. 5D).

A relevant delayed effect of SPEI on the standardized BAI was found for both sexes, but with different timing and magnitude. Positive correlations greater than 0.4 were found for lags between 9 and 36 months in females, with the highest correlation of 0.48 occurring in October, 30 months before tree growth (Fig. 6A). In males, the positive lagged effect of SPEI on standardized BAI was evidenced by correlations greater than 0.4 between 8 and 11 months before tree growth, with the highest correlation of 0.43 occurring in January with a lag of 10 months (Fig. 6B).

Multiple regression models that considered the overall effect of significant climate predictors together were highly statistically significant for both sexes, with climate explaining 30.4% of the standardized BAI variation in male trees and 37.6% in female trees (Table 3). Lagged water availability was the most important climatic predictor of BAI, with SPEI in January with a lag of 10 months being the main predictor in males and SPEI in October with a lag of 30 months in females. Mean temperature in June and potential evapotranspiration in August also showed a positive association with male BAI (Table 3). For females, cloud cover in May and June of the year before growth and mean air temperature in August and September of the year before growth were also significant predictors of BAI (Table 3).

**Discussion**

**Intersex differences in growth rates, Δ^13^C and their relationships**

Males and females of *I. aquifolium* differed in their secondary growth rates, quantified as basal area increment (BAI), with males growing more than females in recent decades. This result contradicts some studies that do not demonstrate different growth rates between the sexes of dioecious woody plants (Martins et al. 2021; Garcia-Barreda et al. 2022). However, our results support other studies that showed higher growth rates in males than females (Montesinos et al. 2006; Nuñez et al. 2008; Cedro and Iszkuło 2011), although the opposite pattern was also found (Rozas et al. 2009; Cattaneo et al. 2013; Desoto et al. 2016). This is not unexpected, as sexual differentiation in growth rates can be site-dependent, with males generally growing more than females in colder and higher elevation locations (Huang et al. 2018; Rozas et al. 2019), and with females often growing at a higher rate in high moisture environments (Ward et al. 2002). In northern Spain, the distribution of *I. aquifolium* extends from sea level to an elevation of 1600 m, and in the Mediterranean mountains occasionally up to 1850 m (Benedí 1997). This means that our studied population at 1400 m is in the upper elevation range of the species. Therefore, it is plausible to hypothesize that males of *I. aquifolium* grow faster than females at our study site given its high elevation, while trees of this species at lower elevations may even show a reversed growth differentiation between the sexes.

The differences in growth rates between the sexes of *I. aquifolium* also changed over time, with females having a higher BAI than males during the period 1900-1930, while from 1980 onwards the trend was reversed and males had a higher BAI than females. Temporal changes in the differences between males and females in their growth patterns have been found in other dioecious plant species (e.g., Rovere et al. 2003), and have been attributed to differences in the timing and cost of reproduction between the sexes, which are expressed differently at different stages of the life cycle (Sánchez Vilas and Pannell 2011; Iszkuło and Boratyński 2011). Furthermore, in a previous study on 3-year-old seedlings, a greater relative growth rate was found in females than in males of *I. aquifolium* (Retuerto et al. 2000). The temporal pattern observed here could be explained by the fact that males of *I. aquifolium* mature earlier and therefore invest more in reproduction (and less in growth) at a younger age than immature females, whose maturation is delayed compared to males (Iszkuło and Boratyński 2011). Males tend to flower earlier in the season than females (Forrest 2014), and although there is less empirical evidence on the life-history transition to maturity, males of some dioecious species have also been found to become reproductively viable at an earlier age than conspecific females (Purrington and Schmitt 1998; Bram and Quinn 2000; Meagher and Delph 2001; Sánchez Vilas and Pannell 2011). Although no data are available for *I. aquifolium*, males of *I. opaca* actually mature earlier than females (Clark and Orton 1967). The earlier age at first reproduction by males has been justified by the fact that females require a longer period of resource accumulation before flowering begins in order to achieve greater reproductive effort due to fruit production (Lloyd and Webb 1977; Purrington and Schmitt 1998; Meagher and Delph 2001). However, adult females of *I. aquifolium* later invest more in reproduction than males (Obeso and Retuerto 2002), which could limit resource allocation for growth and therefore explain that males grow more than females after maturity, as shown in other studies (Obeso et al. 1998; Obeso and Retuerto 2002). Furthermore, the increasing trend in mean temperatures in the study area over the course of the 20^th^ century was particularly pronounced in recent decades (see Fig. 1C). Since the growth of females is more affected by increased temperatures than that of males, this increasing temperature would have a greater impact on females than males.

Carbon isotope discrimination (Δ^13^C) of male and female tree rings did not differ from 1900 to 1930, but from 1930 onwards females had higher Δ^13^C levels, perhaps reflecting a less conservative water use than males. Females are expected to use less conservatively water than males, as has been noted previously (Rozas et al. 2009; Tognetti 2012; Hultine et al. 2013), probably because females have higher resource requirements than males due to fruit production (Obeso 2002; Harris and Pannell 2010; but see Roddy et al. 2019; Midgley and Cramer 2022). Similar results have been found in other dioecious species, such as *Acer negundo* (Dawson and Ehleringer 1993) or *Corema album* (Díaz-Barradas et al. 2018). However, there are also studies that found no differences in carbon isotope discrimination between the sexes (Garcia-Barreda et al. 2022), or if they did exist, they were context dependent (Retuerto et al. 2000). Considering that females of *I. aquifolium* are less conservative in the use of water than males, this could make them more vulnerable to drought than males, especially in drier environments (Dawson and Ehleringer 1993; Retuerto et al. 2000; Hultine et al. 2013). These findings could therefore be important in predicting the potential response of this species to global change. In particular, an increase in drought and/or temperature is expected across the entire range of *I. aquifolium* (IPCC 2023), which could impact the sex ratio of the populations. The sex ratio would become male-biased and thus the risk of population extinction would increase due to the local lack of females (Cruden 2000; Iszkuło et al. 2009; Tognetti 2012; Petry et al. 2016).

We found a negative relationship between BAI and Δ^13^C, suggesting that higher water use efficiency resulted in greater radial growth. Notably, the relationship between BAI and Δ^13^C was stronger in males, indicating a greater physiological connection between growth and carbon isotope discrimination in males than females of *I. aquifolium*. For other tree species, such as the angiosperms *Populus euphratica* (Liu et al. 2014) and *Populus orientalis* (Weiwei et al. 2018), and the gymnosperms *Juniperus thurifera* (Granda et al. 2014) and *Pinus densiflora* (Kwak et al. 2016), long-term increases in BAI concomitant with increases in WUE were found. However, this pattern is not always present (Nock et al. 2011; Wu et al. 2015), suggesting that for some tree species, or for a particular species growing under different environmental conditions (Brienen et al. 2022; Urrutia-Jalabert et al. 2022), it is more difficult to overcome the negative effects of severe constraints by increasing water use efficiency (Heilman et al. 2021). A better understanding of the long-term relationship between growth rates and WUE is crucial to improving our ability to predict how plants might respond to future climate changes (Weiwei et al. 2018; Heilman et al. 2021), particularly for dioecious plant species.

**Influence of sex on the climate response of secondary growth**

Climatic variables significantly influenced the annual growth rates of *I. aquifolium*, with males and females showing different climate sensitivity. Sex-related sensitivity of tree-ring growth to climate have already been reported in a variety of dioecious woody species (Montesinos et al. 2006; Rozas et al. 2009; Iszkuło and Boratyński 2011; Cattaneo et al. 2013; Huang et al. 2018). In some species, females have been found to be more sensitive to certain climatic variables, such as precipitation (Iszkuło et al. 2009; Rozas et al. 2019), but the patterns appear to be complex since sex-specific sensitivity to climate have been found to depend on the environmental context, such as local variations in radiation exposure or elevation (Nuñez et al. 2008; Huang et al. 2018).

Overall, females were more sensitive to the climatic factors of the previous year than males, while males in turn were more influenced by the climatic factors of the current growing year (Fig. 5). Female growth had a positive association with cloud cover but a negative association with mean air temperature and potential evapotranspiration in the previous spring and summer, suggesting that summer water stress would significantly reduce female tree growth in the next growing season. Temperature and cloud cover are negatively correlated (Mendoza et al. 2021), and then opposite effects on tree-ring growth would be expected. Strong negative associations of female growth with past summer temperature have previously been found in several tree species, such as *Taxus baccata* (Cedro and Iszkuło 2011) and *Araucaria araucana* (Rozas et al. 2019). This is likely because warm summers trigger a masting event the following year, which in turn reduces female ring width due to the trade-off between growth and reproduction, resulting in a negative correlation of growth with temperature of the previous summer (Hacket-Pain et al. 2015). In contrast, cloudy and rainy conditions last fall and winter promoted the growth of male trees, for which there was no previous evidence in the literature. More importantly, male growth was positively related to mean air temperature and evapotranspiration in June and August and negatively related to cloud cover in June and August. These results suggest that thermal and lightning conditions that promote high rates of transpiration and photosynthesis in summer are important for the annual growth of male *I. aquifolium* trees. Concurrently, it has been shown that the growth of male *Austrocedrus chilensis* trees is more favoured by high radiation levels than the growth of female trees (Nuñez et al. 2008). The importance of summer conditions on male growth could be related to the fact that flowering in *I. aquifolium* occurs in May-June, and a greater investment in flower production would reduce the available resources that male trees could invest in growth. The differences between sexes in environmental sensitivity found here are complex, but as suggested above these may be related to their differential timing in sink strength as a function of different reproductive phenologies (Iszkuło and Boratyński 2011).

Lagged correlations between radial growth of *I. aquifolium* and water availability, estimated by cumulative SPEI during the last 36 months, showed that males had a greater sensitivity to drought in a 10-months period, while females showed greater sensitivity to drought in a 30-months period. These extremely delayed drought legacies can be observed in trees growing in arid or semiarid environments under chronic water deficit (Peltier and Ogle 2019; Rozas et al. 2021). However, our results revealed that female *I. aquifolium* trees growing in a temperate climate are also more influenced by past climatic conditions than males, suggesting that female trees have greater drought memory than male trees (Peltier and Ogle 2023). SPEI sensitivity was greater in females than in males (0.48 F vs. 0.43 M; Fig. 6), although female growth was also highly correlated with SPEI ranging from February to June with a lag of 21-26 months, as well as during February-March with a lag of 12-13 months. This was likely because resource budgets and weather are involved in the functional mechanisms that drive vegetative growth and reproduction, and both processes would then be coupled in the long term, particularly for females (Montesinos et al. 2006; Pearse et al. 2016). Interestingly, Garcia-Barreda et al. (2022) showed that the lagged sex-specific effects of drought stress on growth can be also species-specific, with the medium-term effect of water availability being larger in males in some species but larger in females in others.

Considering that trees of both sexes undergo the same environmental fluctuations, differences may be due to functional traits related to reproduction since as pointed above, adult female trees of this species usually incur higher costs of reproduction than adult male trees (Obeso 1997; Obeso and Retuerto 2002) . Different climatic affinities resulting from sexual differentiation would ultimately indicate a compensatory functional response of growth (DeClerk et al. 2006; Tornos-Estupiña et al. 2023). However, for populations growing at the rear or dry edge of the distribution range the disadvantages may easily outweigh the advantages, as the trend of rising temperatures is constraining tree growth (Shestakova et al. 2016). High dependence on climate poses a high risk of growth changes during and in the years immediately following drought episodes (Peltier and Ogle 2020), which can lead to tree malfunction and tree death (Adams et al. 2017). In general, males exhibit a more efficient water use and are generally less sensitive to increased drought than co-occurring females (Hultine et al. 2016, 2018), therefore a male-biased sex ratio is possible in the future in a significant number of populations.

**Conclusions**

We found that sex differences in secondary growth rates were unstable over time, with males outperforming females in both radial growth rates and water use efficiency in recent decades. Additionally, males showed a stronger relationship between water use efficiency and growth rates than females, which is likely due to the higher reproductive effort of females compared with males. Furthermore, our results showed a sex-dependent response of secondary growth to climatic conditions, with summer water stress significantly reducing the growth of female trees in the next growing season. In contrast, male tree growth was primarily favoured by cloudy and rainy conditions last fall and winter, combined with low cloud cover and warm conditions in summer. This study is the first to report sex-dependent lagged correlations between *I. aquifolium* radial growth and water availability, with the strongest relationship between tree growth and cumulative water availability at 30 months in females and at 10 months in males. Therefore, we suggest that male growth depends on water availability on short time scales, while female growth is related to cumulative water availability over medium to long time scales. It is likely that these differences between the sexes arise because the functional mechanisms that drive vegetative growth and reproduction depend on past weather, which determines current resource budgets, and both processes are more strongly coupled in the long term in females than in males.

**Acknowledgements**

The authors thank Brígida Fernández and Sergio R. Roiloa for continuous help in field work, and Brígida Fernández also for intensive laboratory work. Thanks also to Xabier Santiso for some exploratory data work, to the administrators of the Ancares Nature Reserve for allowing field sampling and their support, and to Dr Adam Roddy and an anonymous reviewer for helpful comments to an earlier version of the manuscript.

**Funding**

This research was supported by the Spanish Ministry of Science and Technology (grant BOS2002-00714).

**Data availability**

The data that support the findings of this study are available from the authors upon reasonable request.

**References**

**Adams HD, Zeppel MJB, Anderegg WRL, Hartmann H, Landhäusser SM, Tissue DT, Huxman TE, Hudson PJ, Franz TE, Allen CD, Anderegg LDL, Barron-Gafford GA, Beerling DJ, Breshears DD, Brodribb TJ, Bugmann H, Cobb RC, Collins AD, Dickman LT, Duan H, Ewers BE, Galiano L, Galvez DA, Garcia-Forner N, Gaylord ML, Germino MJ, Gessler A, Hacke UG, Hakamada R, Hector A, Jenkins MW, Kane JM, Kolb TE, Law DJ, Lewis JD, Limousin JM, Love DM, Macalady AK, Martínez-Vilalta J, Mencuccini M, Mitchell PJ, Muss JD, O’Brien MJ, O’Grady AP, Pangle RE, Pinkard EA, Piper FI, Plaut JA, Pockman WT, Quirk J, Reinhardt K, Ripullone F, Ryan MG, Sala A, Sevanto S, Sperry JS, Vargas R, Vennetier M, Way DA, Xu C, Yepez EA, McDowell NG**. **2017**. A multi-species synthesis of physiological mechanisms in drought-induced tree mortality. *Nature Ecology and Evolution* **1**: 1285–1291.

**Akaike H**. **1973**. Information theory as an extension of the maximum likelihood principle. In: Petrov BN,, In: Csaki F, eds. *Second International Symposium on Information Theory*. Budapest: Akadmiai Kiado, 267–281.

**Barrett SCH, Hough J**. **2013**. Sexual dimorphism in flowering plants. *Journal of Experimental Botany* **64**: 67–82.

**Barton K**. **2022**. MuMIn: Multi-Model Inference. R package version 1.46.0. https://CRAN.R-project.org/package=MuMIn.

**Beguería S, Vicente-Serrano SM**. **2017**. SPEI: Calculation of the Standardised Precipitation-Evapotranspiration Index. R package version 1.7. https://CRAN.R-project.org/package=SPEI.

**Benedí C**. **1997**. Aquifoliaceae. In: Castroviejo S,, In: Aedo C,, In: Laínz M,, In: Muñoz Garmendia F,, In: Nieto Feliner G,, In: Paiva J,, In: Benedí C, eds. *Flora Ibérica. Vol. III. Haloragaceae-Euphorbiaceae*. Madrid: Real Jardín Botánico, CSIC, 182–185.

**Benner R, Fogel ML, Sprague EK, Hodson RE**. **1987**. Depletion of ^13^C in lignin and its implications for stable carbon isotope studies. *Nature* **329**: 708–710.

**Biondi F, Qeadan F**. **2008**. A theory-driven approach to tree-ring standardization: defining the biological trend from expected basal area increment. *Tree-Ring Research* **64**: 81–96.

**Bram MR, Quinn JA**. **2000**. Sex expression, sex-specific traits, and the effects of salinity on growth and reproduction of *Amaranthus cannabinus* (Amaranthaceae), a dioecious annual. *American Journal of Botany* **87**: 1609–1618.

**Brienen R, Helle G, Pons T, Boom A, Gloor M, Groenendijk P, Clerici S, Leng M, Jones C**. **2022**. Paired analysis of tree ring width and carbon isotopes indicates when controls on tropical tree growth change from light to water limitations. *Tree Physiology* **42**: 1131–1148.

**Brooks JR, Flanagan LB, Ehleringer JR**. **1998**. Responses of boreal conifers to climate fluctuations: indications from tree-ring widths and carbon isotope analyses. *Canadian Journal of Forest Research* **28**: 524–533.

**Burnham KP, Anderson DR**. **2002**. *Model selection and multimodels inference: a practical information-theoretic approach.* New York, USA: Springer.

**Campelo F, García-González I, Nabais C**. **2012**. detrendeR – A Graphical User Interface to process and visualize tree-ring data using R. *Dendrochronologia* **30**: 57–60.

**De Candolle A**. **1855**. *Geographie Botanique Raisonee*. Paris: Library V. Masson.

**Cattaneo N, Pahr N, Fassola H, Leporati J, Bogino S**. **2013**. Sex-related, growth–climate association of *Araucaria angustifolia* in the neotropical ombrophilous woodlands of Argentina. *Dendrochronologia* **31**: 147–152.

**Cedro A, Iszkuło G**. **2011**. Do females differ from males of European yew (*Taxus baccata* L.) in dendrochronological analysis? *Tree-Ring Research* **67**: 3–11.

**Clark RB, Orton ER**. **1967**. Sex ratio in *Ilex opaca* Ait. *HortScience* **2**: 115.

**Cruden RW**. **2000**. Pollen grains: Why so many? *Plant Systematics and Evolution* **222**: 143–165.

**Damesin C, Rambal S, Joffre R**. **1997**. Between-tree variations in leaf ∂13C of *Quercus pubescens* and *Quercus ilex* among Mediterranean habitats with different water availability. *Oecologia* **111**: 26–35.

**Damesin C, Rambal S, Joffre R**. **1998**. Seasonal and annual changes in leaf δ^13^C in two co-occurring Mediterranean oaks: relations to leaf growth and drought progression. *Functional Ecology* **12**: 778–785.

**Dawson TE, Bliss LC**. **1989**. Patterns of water use and the tissue water relations in the dioecious shrub, Salix arctica: the physiological basis for habitat partitioning between the sexes. *Oecologia* **79**: 332–343.

**Dawson TE, Ehleringer JR**. **1993**. Gender-specific physiology, carbon isotope discrimination, and habitat distribution in boxelder, Acer negundo. *Ecology* **74**: 798–815.

**Dawson TE, Ward JK, Ehleringer JR**. **2004**. Temporal scaling of physiological responses from gas exchange to tree rings: a gender-specific study of Acer negundo (Boxelder) growing under different conditions. *Functional Ecology* **18**: 212–222.

**DeClerk FAJ, Barbour MG, Sawyer JO**. **2006**. Species richness and stand stability in conifer forests of the Sierra Nevada. *Ecology* **87**: 2787–2799.

**Desoto L, Olano JM, Rozas V**. **2016**. Secondary growth and carbohydrate storage patterns differ between sexes in *Juniperus thurifera*. *Frontiers in Plant Science* **7**: 173698.

**Díaz-Barradas MC, Zunzunegui M, Correia O, Ain-Lhout F, Esquivias MP, Álvarez-Cansino L**. **2018**. Gender dimorphism in *Corema album* across its biogeographical area and implications under a scenario of extreme drought events. *Environmental and Experimental Botany* **155**: 609–618.

**Dupouey J ‐L, Leavitt S, Choisnel E, Jourdain S**. **1993**. Modelling carbon isotope fractionation in tree rings based on effective evapotranspiration and soil water status. *Plant, Cell and Environment* **16**: 939–947.

**Eckes-Shephard AH, Ljungqvist FC, Drew DM, Rathgeber CBK, Friend AD**. **2022**. Wood formation modeling – a research review and future perspectives. *Frontiers in Plant Science* **13**: 837648.

**Ehleringer JR**. **1993**. Carbon and water relations in desert plants: an isotopic perspective. In: Ehleringer, J. R.; Hall, A.E.; Farquhar GD, ed. *Stable Isotopes and Plant Carbon/Water Relations*. San Diego: Academic Press, 155–172.

**Farquhar GDD, Ehleringer JR, Hubick KT**. **1989**. Carbon isotope discrimination and photosynthesis. *Annual Review of Plant Physiology* **40**: 503–537.

**Farquhar GD, O’Leary MH, Berry JA**. **1982**. On the relationship between carbon isotope discrimination and the intercellular carbon dioxide concentration in leaves. *Australian Journal of Plant Physiology* **9**: 121–137.

**Ferrio JP, Florit A, Vega A, Serrano L, Voltas J**. **2003**. Δ^13^C and tree-ring width reflect different drought responses in *Quercus ilex* and *Pinus halepensis*. *Oecologia* **137**: 512–518.

**Fleck I, Grau D, Sanjosé M, Vidal D**. **1996**. Carbon isotope discrimination in *Quercus ilex* resprouts after fire and tree-fell. *Oecologia* **105**: 286–292.

**Forrest JRK**. **2014**. Plant size, sexual selection, and the evolution of protrandry in dioecious plants. *The American Naturalist* **184**: 338–351.

**Garcia-Barreda S, Sangüesa-Barreda G, García-González MD, Camarero JJ**. **2022**. Sex and tree rings: Females neither grow less nor are less water-use efficient than males in four dioecious tree species. *Dendrochronologia* **73**: 125944.

**Gessler A, Ferrio JP, Hommel R, Treydte K, Werner RA, Monson RK**. **2014**. Stable isotopes in tree rings: towards a mechanistic understanding of isotope fractionation and mixing processes from the leaves to the wood. *Tree Physiology* **34**: 796–818.

**Granda E, Rossatto DR, Camarero JJ, Voltas J, Valladares F**. **2014**. Growth and carbon isotopes of Mediterranean trees reveal contrasting responses to increased carbon dioxide and drought. *Oecologia* **174**: 307–317.

**Guitián J, Bermejo T**. **2006**. Dynamics of plant–frugivore interactions: a long-term perspective on holly–redwing relationships in northern Spain. *Acta Oecologica* **30**: 151–160.

**Hacket-Pain AJ, Friend AD, Lageard JGA, Thomas PA**. **2015**. The influence of masting phenomenon on growth–climate relationships in trees: explaining the influence of previous summers’ climate on ring width. *Tree Physiology* **35**: 319–330.

**Harris I, Osborn TJ, Jones P, Lister D**. **2020**. Version 4 of the CRU TS monthly high-resolution gridded multivariate climate dataset. *Scientific Data 2020 7:1* **7**: 1–18.

**Harris MS, Pannell JR**. **2010**. Canopy seed storage is associated with sexual dimorphism in the woody dioecious genus Leucadendron. *Journal of Ecology* **98**: 509–515.

**Hartman G, Danin A**. **2010**. Isotopic values of plants in relation to water availability in the Eastern Mediterranean region. *Oecologia* **162**: 837.

**Hebbali A**. **2020**. olsrr. Tools for building OLS Regression Models. R package version 0.5.3.

**Heilbuth J**. **2000**. Lower species richness in dioecious clades. *American Naturalist* **156**: 221–241.

**Heilman KA, Trouet VM, Belmecheri S, Pederson N, Berke MA, McLachlan JS**. **2021**. Increased water use efficiency leads to decreased precipitation sensitivity of tree growth, but is offset by high temperatures. *Oecologia* **197**: 1095.

**Huang K, Liao Y, Dong T, Yang Y, He J, Huan H, Zhang Q, Xu X**. **2018**. Sex-specific responses of tree-ring growth to climate in the dioecious tree *Populus cathayana*. *Journal of Plant Ecology* **11**: 771–779.

**Hultine KR, Burtch KG, Ehleringer JR**. **2013**. Gender specific patterns of carbon uptake and water use in a dominant riparian tree species exposed to a warming climate. *Global Change Biology* **19**: 3390–3405.

**Hultine KR, Bush SE, Ward JK, Dawson TE**. **2018**. Does sexual dimorphism predispose dioecious riparian trees to sex ratio imbalances under climate change? *Oecologia* **187**: 921–931.

**Hultine KR, Grady KC, Wood TE, Shuster SM, Stella JC, Whitham TG**. **2016**. Climate change perils for dioecious plant species. *Nature Plants* **2**: 16109.

**IPCC**. **2023**. *Climate Change 2023: Synthesis Report. Contribution of working groups I, II and III to the Sixth Assessment Report of Untergovernmental Panel on Climate Change* (H Lee and J Romero, Eds.). Geneva, Switzerland.

**Iszkuło G, Boratyński A**. **2011**. Initial period of sexual maturity determines the greater growth rate of male over female in the dioecious tree Juniperus communis subsp. communis. *Acta Oecologica* **37**: 99–102.

**Iszkuło G, Jasińska AK, Giertych MJ, Boratyński A**. **2009**. Do secondary sexual dimorphism and female intolerance to drought influence the sex ratio and extinction risk of *Taxus baccata*? *Plant Ecology* **200**: 229–240.

**Iversen J**. **1944**. *Viscum*, *Hedera* and *Ilex* as climatic indicators. *Geol. For. Stockh. Forh.* **66**: 463–483.

**Juvany M, Munné-Bosch S**. **2015**. Sex-related differences in stress tolerance in dioecious plants: a critical appraisal in a physiological context. *Journal of Experimental Botany* **66**: 6083–6092.

**Körner C, Farquhar GD, Roksandic Z**. **1988**. A global survey of carbon isotope discrimination in plants from high altitude. *Oecologia* **74**: 623–632.

**Korol RL, Kirschbaum MUF, Farquhar GD, Jeffreys M**. **1999**. Effects of water status and soil fertility on the C-isotope signature in *Pinus radiata*. *Tree Physiology* **19**: 551–562.

**Kwak JH, Lim SS, Lee KS, Viet HD, Matsushima M, Lee KH, Jung K, Kim HY, Lee SM, Chang SX, Choi WJ**. **2016**. Temperature and air pollution affected tree ring δ^13^C and water-use efficiency of pine and oak trees under rising CO_2_ in a humid temperate forest. *Chemical Geology* **420**: 127–138.

**Leavitt SW, Danzer SR**. **1993**. Method for batch processing small wood samples to holocellulose for stable-carbon isotope analysis. *Analytical Chemistry* **65**: 87–89.

**Liu X, Wang W, Xu G, Zeng X, Wu G, Zhang X, Qin D**. **2014**. Tree growth and intrinsic water-use efficiency of inland riparian forests in northwestern China: evaluation via δ^13^C and δ^18^O analysis of tree rings. *Tree Physiology* **34**: 966–980.

**Lloyd DG, Webb CJ**. **1977**. Secondary sex characters in plants. *Botanical Review* **43**: 177–216.

**Martins VF, Bispo RLB, de Paula Loiola P**. **2021**. A case of gender equality: absence of sex-related costs in a dioecious tropical forest tree species. *Plant Ecology* **222**: 275–288.

**Meagher TR, Delph LF**. **2001**. Individual flower demography, floral phenology and floral display size in *Silene latifolia*. *Evolutionary Ecology Research* **3**: 845–860.

**Mendoza V, Pazos M, Garduño R, Mendoza B**. **2021**. Thermodynamics of climate change between cloud cover, atmospheric temperature and humidity. *Scientific Reports* **11**: 21244.

**Midgley JJ, Cramer MD**. **2022**. Unequal allocation between male versus female reproduction cannot explain extreme vegetative dimorphism in *Aulax species* (Cape Proteaceae). *Scientific Reports* **12**: 1–7.

**Milla R, Castro-Díez P, Maestro-Martínez M, Montserrat-Martí G**. **2006**. Costs of reproduction as related to the timing of phenological phases in the dioecious shrub *Pistacia lentiscus* L. *Plant Biology* **8**: 103–111.

**Montesinos D, de Luís M, Verdú M, Raventós J, García-Fayos P**. **2006**. When, how and how much: Gender-specific resource-use strategies in the dioecious tree *Juniperus thurifera*. *Annals of Botany* **98**: 885–889.

**Nock CA, Baker PJ, Wanek W, Leis A, Grabner M, Bunyavejchewin S, Hietz P**. **2011**. Long-term increases in intrinsic water-use efficiency do not lead to increased stem growth in a tropical monsoon forest in western Thailand. *Global Change Biology* **17**: 1049–1063.

**Nuñez CI, Ecotono L, Regional C, Bariloche U, Nacional U, Nuñez MA, Kitzberger T**. **2008**. Sex-related spatial segregation and growth in a dioecious conifer along environmental gradients in northwestern Patagonia. *Ecoscience* **15**: 73–80.

**Obeso JR**. **1997**. Costs of reproduction in *Ilex aquifolium*: effects at tree, branch and leaf levels. *Journal of Ecology* **85**: 159–166.

**Obeso JR**. **2002**. The cost of reproduction in plants. *New Phytologist* **155**: 321–348.

**Obeso JR, Alvarez-Santullano M, Retuerto R**. **1998**. Sex ratios, size distributions, and sexual dimorphism in the dioecious tree *Ilex aquifolium* (Aquifoliaceae). *American Journal of Botany* **85**: 1602–1608.

**Obeso JR, Retuerto R**. **2002**. Dimorfismo sexual en el acebo, Ilex aquifolium: ¿coste de la reproducción, selección sexual o diferenciación fisiológica? Sexual dimorphism in holly Ilex aquifolium: cost of reproduction, sexual selection or physiological differentiation? *Revista Chilena de Historia Natural* **75**: 67–77.

**Pannell JR, Jordan CY**. **2022**. Evolutionary transitions between hermaphroditism and dioecy in animals and plants. *Annual Review of Ecology and Systematics* **53**: 183–201.

**Pearse IS, Koenig WD, Kelly D**. **2016**. Mechanisms of mast seeding: resources, weather, cues, and selection. *New Phytologist* **212**: 546–562.

**Peltier DMP, Ogle K**. **2019**. Legacies of more frequent drought in ponderosa pine across the western United States. *Global Change Biology* **25**: 3803–3816.

**Peltier DMP, Ogle K**. **2020**. Tree growth sensitivity to climate is temporally variable. *Ecology Letters* **23**: 1561–1572.

**Peltier DMP, Ogle K**. **2023**. Still recovering or just remembering? To understand drought legacies, modelling choices matter. *Journal of Ecology* **111**: 1170–1173.

**Peterken GF, Lloyd PS**. **1967**. *Ilex aquifolium* L. *Journal of Ecology* **55**: 841–858.

**Petry WK, Soule JD, Iler AM, Chicas-Mosier A, Inouye DW, Miller TEX, Mooney KA**. **2016**. Sex-specific responses to climate change in plants alter population sex ratio and performance. *Science* **353**: 69–71.

**Pollo CJ, Robles L, Seijas JM, García-Miranda A, Otero R**. **2005**. Trends in the abundance of Cantabrian Capercaillie *Tetrao urogallus cantabricus*  at leks on the southern slope of the Cantabrian Mountains, north-west Spain. *Bird Conservation International* **15**: 397–409.

**Purrington CB, Schmitt J**. **1998**. Consequences of sexually dimorphic timing of emergence and flowering in Silene latifolia. *Journal of Ecology* **86**: 397–404.

**R Core Team**. **2022**. R: A language and environment for statistical computing.

**Renner SS**. **2014**. The relative and absolute frequencies of angiosperm sexual systems: Dioecy, monoecy, gynodioecy, and an updated online database. *American Journal of Botany* **101**: 1588–1596.

**Retuerto R, Fernandez-Lema B, Obeso JR**. **2006**. Changes in photochemical efficiency in response to herbivory and experimental defoliation in the dioecious tree *Ilex aquifolium*. *International Journal of Plant Sciences* **167**: 279–289.

**Retuerto R, Lema BF, Roiloa SR, Obeso JR**. **2000**. Gender, light and water effects in carbon isotope discrimination, and growth rates in the dioecious tree *Ilex aquifolium*. *Functional Ecology* **14**: 529–537.

**Retuerto R, Sánchez Vilas J, Varga S**. **2018**. Sexual dimorphism in response to stress. *Environmental and Experimental Botany* **146**: 1–4.

**Roddy AB, van Blerk JJ, Midgley JJ, West AG**. **2019**. Ramification has little impact on shoot hydraulic efficiency in the sexually dimorphic genus Leucadendron (Proteaceae). *PeerJ* **7**: e6835.

**Rovere AE, Aizen MA, Kitzberger T**. **2003**. Growth and climatic response of male and female trees of *Austrocedrus chilensis*, a dioecious conifer from the temperate forests of southern South America. *Ecoscience* **10**: 195–203.

**Rozas V, DeSoto L, Olano JM**. **2009**. Sex-specific, age-dependent sensitivity of tree-ring growth to climate in the dioecious tree *Juniperus thurifera*. *New Phytologist* **182**: 687–697.

**Rozas V, García-Cervigón AI, García-Hidalgo M, Rodríguez-García E, Olano JM**. **2021**. Living on the edge: Legacy of water availability on *Tetraclinis articulata* secondary growth under semiarid conditions in Morocco. *Dendrochronologia* **68**: 125853.

**Rozas V, Le Quesne C, Rojas-Badilla M, González-Reyes Á, Donoso S, Olano JM**. **2019**. Climatic cues for secondary growth and cone production are sex-dependent in the long-lived dioecious conifer *Araucaria araucana*. *Agricultural and Forest Meteorology* **274**: 132–143.

**Sánchez Vilas J, Pannell JR**. **2011**. Sexual dimorphism in resource acquisition and deployment: both size and timing matter. *Annals of Botany* **107**: 119–126.

**Saurer M, Siegenthaler U**. **1989**. ^13^C/^12^C isotope ratios in trees are sensitive to relative humidity. *Dendrochronologia* **7**: 9–13.

**Schweingruber FH, Baas P**. **2011**. *Anatomy of European woods: an atlas for the identification of European trees, shrubs and dwarf shrubs.* Verlag Kessel.

**Seibt U, Rajabi A, Griffiths H, Berry JA**. **2008**. Carbon isotopes and water use efficiency: sense and sensitivity. *Oecologia* **155**: 441–454.

**Shestakova TA, Gutiérrez E, Kirdyanov A V., Camarero JJ, Génova M, Knorre AA, Linares JC, De Dios VR, Sánchez-Salguero R, Voltas J**. **2016**. Forests synchronize their growth in contrasting Eurasian regions in response to climate warming. *Proceedings of the National Academy of Sciences of the United States of America* **113**: 662–667.

**Da Silveira L, Sternberg L**. **1989**. Oxygen and hydrogen isotope measurments in plant cellulose analysis. In: Linokens HF,, In: Jackson F, eds. *Modern Methods of Plant Analysis, Vol. 10. Plant Fibers*. Springer-Verlag, Heidelberg, 89–99.

**Tans PP, De Jong AFM, Mook WG**. **1978**. Chemical pretreatment and radial flow of ^14^C in tree rings. *Nature* **271**: 234–235.

**Tognetti R**. **2012**. Adaptation to climate change of dioecious plants: does gender balance matter? *Tree Physiology* **32**: 1321–1324.

**Tornos-Estupiña L, Hernández-Alonso H, Madrigal-González J, Rodrigues A, Silla F**. **2023**. Species-specific growth responses to local and regional climate variability indicate the presence of a diversity portfolio effect in mediterranean tree assemblages. *Agricultural and Forest Meteorology* **341**: 109665.

**Urrutia-Jalabert R, Barichivich J, Szejner P, Rozas V, Lara A**. **2022**. Ecophysiological responses of *Nothofagus obliqua* forests to recent climate drying across the Mediterranean-Temperate biome transition in south-central Chile. *Journal of Geophysical Research. Biogeosciences* **128**: e2022JG007293.

**Vamosi JC, Otto SP, Barrett SCH**. **2003**. Phylogenetic analysis of the ecological correlates of dioecy in angiosperms. *Journal of Evolutionary Biology* **16**: 1006–1018.

**Vicente-Serrano SM, Beguería S, López-Moreno JI**. **2010**. A multiscalar drought index sensitive to global warming: the Standardized Precipitation Evapotranspiration Index. *Journal of Climate* **23**: 1696–1718.

**Ward HM**. **1904**. *Trees: A handbook of forest-botany for the woodlands and the laboratory* (AE Shipley, Ed.). Cambridge University Press.

**Ward JK, Dawson TE, Ehleringer JR**. **2002**. Responses of Acer negundo genders to interannual differences in water availability determined from carbon isotope ratios of tree ring cellulose. *Tree Physiology* **22**: 339–346.

**Warren CR, McGrath JF, Adams MA**. **2001**. Water availability and carbon isotope discrimination in conifers. *Oecologia* **127**: 476–486.

**Weiwei LU, Xinxiao YU, Guodong JIA, Hanzhi LI, Ziqiang LIU**. **2018**. Responses of intrinsic water-use efficiency and tree growth to climate change in semi-arid areas of North China. *Scientific Reports* **8**: 1–10.

**Wu G, Liu X, Chen T, Xu G, Wang W, Zeng X, Zhang X**. **2015**. Elevation-dependent variations of tree growth and intrinsic water-use efficiency in Schrenk spruce (*Picea schrenkiana*) in the western Tianshan Mountains, China. *Frontiers in Plant Science* **6**: 1–12.

**Table 1**. Characteristics of the studied male and female *Ilex aquifolium* trees, individual secondary growth rates (BAI) and carbon isotope discrimination (∆^13^C) series from Cavana Vella, Sierra de Ancares, Lugo, Spain.

|  | Male | Female |
| --- | --- | --- |
|  |  |  |
|  |  |  |
| Number of trees | 16 | 14 |
| Number of cores | 43 | 32 |
| Mean (±SE) stem diameter (cm) | 18.5 ± 0.7 | 18.5 ± 0.9 |
| Stem diameter range (cm) | 14.2–23.1 | 13.4–23.3 |
| Mean (±SE) tree age (yr) | 93.9 ± 7.4 | 99.1 ± 9.9 |
| Tree age range (yr) | 37–140 | 54–142 |
| Mean (±SE) BAI (cm^2^ yr^-1^) ^(a)^ | 3.16 ± 0.23 | 2.52 ± 0.15 |
| BAI range (cm^2^ yr^-1^) ^(a)^ | 1.47–4.39 | 1.67–3.91 |
| Mean (±SE) ∆^13^C (‰)^(b)^ | 17.51 ± 0.13 | 18.15 ± 0.16 |
| ∆^13^C range (‰)^(b)^ | 16.92–18.26 | 17.45–19.04 |
|  |  |  |

^(a)^ From annual BAI series in the period 1942–2001.

^(b)^ From ∆^13^C quantified in 5-yr intervals in the period 1903–2001.

**Table 2**. Summary of the best LM models for secondary growth (BAI) and carbon isotope discrimination (∆^13^C) during the 1900-2000 period, quantifying the individual response of *Ilex aquifolium* and showing the effects of time (Pentad), sex, ecological memory (BAI_p-1_ and ∆^13^C_p-1_) and size (DBH) as covariates. The model type III *F*-statistic with the associated *p*-value are shown. Significant effects (*p* < 0.05) are highlighted in bold. Best models enhanced their null model counterpart in ∆AICc _null - best_ = 528.8 (BAI) and 463.0 (∆^13^C) units.

| Best model | Model *r*_adj_^2^ | Model *F* | Model *p* | Predictor variable | *β* | CI | *p*-value |
| --- | --- | --- | --- | --- | --- | --- | --- |
|  |  |  |  |  |  |  |  |
|  |  |  |  |  |  |  |  |
| BAI | 0.682 | 200.5 | <0.001 | Pentad | 0.00 | –0.00 – 0.00 | 0.177 |
|  |  |  |  | Sex [Male] | –1.00 | -1.00 – –0.99 | **<0.001** |
|  |  |  |  | BAI p-1 | 0.20 | 0.18 – 0.23 | **<0.001** |
|  |  |  |  | DBH | 0.01 | 0.00 – 0.02 | **0.010** |
|  |  |  |  | Pentad × Sex [Male] | 0.00 | 0.00 – 0.01 | **<0.001** |
| Δ^13^C | 0.752 | 254.2 | <0.001 | Pentad | –0.00 | –0.00 – 0.00 | 0.330 |
|  |  |  |  | Sex [Male] | 7.59 | 0.31 – 14.88 | **0.041** |
|  |  |  |  | Δ^13^C p-1 | 0.76 | 0.69 – 0.83 | **<0.001** |
|  |  |  |  | Pentad × Sex [Male] | –0.00 | –0.01 – –0.00 | **0.036** |
|  |  |  |  |  |  |  |  |

**Table 3**. Statistics of the stepwise multiple linear regression models for standardized BAI chronologies of male and female *Ilex aquifolium* trees with the significant climatic predictors over the period 1942–2001 (n = 60).

| Sex | Model *r*_adj_^2^ | Model *F* | Model *p* | Predictor variable ^(*)^ | *β* | *t*-value | *p*-value |
| --- | --- | --- | --- | --- | --- | --- | --- |
|  |  |  |  |  |  |  |  |
|  |  |  |  |  |  |  |  |
| Male | 0.304 | 9.58 | <0.001 | SPEI11 Jan | 0.375 | 3.43 | 0.001 |
|  |  |  |  | Tmed Jun | 0.272 | 2.35 | 0.022 |
|  |  |  |  | PET Aug | 0.240 | 2.06 | 0.044 |
| Female | 0.376 | 12.83 | <0.001 | SPEI30 Oct | 0.340 | 3.18 | 0.002 |
|  |  |  |  | Cld MayJun(-1) | 0.325 | 3.06 | 0.003 |
|  |  |  |  | Tmed AugSep(-1) | –0.309 | –2.78 | 0.004 |
|  |  |  |  |  |  |  |  |

^(*)^ Code interpretation for climate predictors: Tmed, mean temperature; Cld, cloudiness; PET, potential evapotranspiration; SPEI, standardized precipitation evapotranspiration index. Jan to Dec means the months of the year, from January to December; (-1), year before tree growth. Two months indicate that the average of two adjacent months has been calculated. SPEI11 and SPEI30 refer to the accumulated SPEI of the last 11 and 30 months, respectively.

**Figure Legends**

**Figure 1**. Location of the study area in the Sierra de Ancares, Galicia, north-western Spain (A). Climatic diagram of the Sierra de Ancares for the period 1901–2000 (B). Maximum (Tmax), mean (Tmean) and minimum (Tmin) monthly temperatures as well total monthly precipitation (Prec) series are displayed along with mean annual temperature (T) and total annual precipitation (P). Temporal variation of the mean annual temperature in the period 1901-2000, with its linear fit, r^2^ and *p*-values (C) are shown.

**Figure 2**. (A) Annual variation in basal area increment (BAI, cm^2^ yr^-1^) for *Ilex aquifolium* males and females, and variation at 5-yr intervals of (B) mean (± SE) BAI and (C) mean (± SE) carbon isotope discrimination (∆^13^C, ‰) for males and females.

**Figure 3**. Adjusted linear predictions of the Year * Sex interaction term from BAI (A) and ∆^13^C (B) best models (Table 2). Shaded area shows confidence intervals at 95% level.

**Figure 4**. Relationships between mean basal area increment (BAI, cm^2^ yr^-1^) and carbon isotope discrimination (∆^13^C, ‰) at 5-yr intervals for males and females. The obtained linear fits, their coefficients of determination and their statistical significance are shown.

**Figure 5**. Correlations of annual standardized basal area increments with the monthly climate variables (A) mean temperature, (B) precipitation, (C) cloud cover and (D) potential evapotranspiration for males (black bars) and females (grey bars) over the period 1942-2001. Dashed lines indicate *p* < 0.05 and dotted lines indicate *p* < 0.01 significance levels. Jan to Dec refers to the months of the year, from January to December; (-1) indicates the year before the trees grew.

**Figure 6**. Correlation (r) of the standardized BAI chronologies of (A) female and (B) male *Ilex aquifolium* trees in Sierra de Ancares explained by monthly Standardized Precipitation–Evaporation Index (SPEI) series at different time scales for the period 1942–2001. The analyses were calculated from September of the previous year to December of the current growth year (previous year months depicted in capitals). Insets in the graphs (dotted lines and black diamonds) represent the month and time lag at which the maximum correlation occurs.
