# Supplementary material for: Differential growth rate, water-use efficiency and climate sensitivity between males and females of Ilex aquifolium in north-western Spain
Source: Ann Bot. 2024 Aug 7;135(1-2):357–70. doi: 10.1093/aob/mcae126 (PMC11805936; doi:10.1093/aob/mcae126)
Supplement: mcae126_suppl_Supplementary_Material [file mcae126_suppl_supplementary_material.zip › aob-24204-s01.docx]

**Dr Julia Sánchez Vilas**

Monday, 22^nd^ July 2024

Please find herewith a resubmitted version of our manuscript (ID: 24204): “*Differential growth rate, water use efficiency and climate sensitivity between males and females of Ilex aquifolium in north-western Spain*” authored by Sánchez Vilas, Hernández-Alonso, Rozas and Retuerto.

As detailed below, we have modified the manuscript in accordance with the comments received and believe that the resubmitted version has improved as a result.

Please, see below the point-by-point reply to the comments. We have uploaded in the supplementary material a version of the revised manuscript with the changes highlighted using the “Track Changes” feature of MS.

We trust that our manuscript will now be acceptable for publication.

Looking forward to hearing from you.

Sincerely,

Julia Sánchez Vilas

**RESPONSE TO THE COMMENTS:**

Dear Dr Sanchez Vilas,

Thank you for sending your manuscript entitled "Differential growth rate, water use efficiency and climate sensitivity between males and females of *Ilex aquifolium* in north-western Spain" to Annals of Botany. It has been evaluated by a referee (whose comments are given at the end of this letter) and also by the Handling Editor, Dr Adam Roddy, whose views are as follows:

Authors: We are very grateful to Dr Adam Roddy and the reviewer for the useful and constructive comments given to our manuscript. We have addressed these point-by-point as indicated below– with changes highlighted in the manuscript.

---

I sincerely apologize to the authors for how long it has taken to review this manuscript. It took a surprisingly long time to find reviewers who would accept the review, and then one of them disappeared and quit responding. Admittedly, I was not paying too much attention, as I was in the process of moving to a different city, and so I am only now catching up. Given the delay in finding external reviewers, I have chosen to review the paper myself, in addition to one external review.

As you will see below, both the reviewer and I like this manuscript quite a lot. The methods and analyses are robust, generally well-articulated, and well-reasoned. I am particularly happy to see that the manuscript is fair in its description of how existing data do or do not support ideas: namely, the manuscript states explicitly that the costs of reproduction are typically assumed to be high. However, both the reviewer and I have some (relatively small) changes that should be made in a future version of the manuscript. In addition, in my comment to line 524, I ask about other (perhaps unmeasured) variables that might be influencing these dynamics, such as the costs of reproduction between the sexes and interannually. While there may not be data to discuss about this, I do think it is worth explaining how these other variables could influence the patterns.

63 some relevant citations: Dawson and Ehleringer (1993, Ecology) and Dawson et al. (2004, Functional Ecology)

Authors: Citations added.

84 I don't understand what the "as" is doing for the rest of this sentence...?

Authors: ‘as’ was misplaced and delete from the sentence.

101 We typically don't like speaking of "proving" responses or hypotheses. I suggest making this statement more agnostic to outcome.

Authors: This statement has been changed as suggested.

367 "area" is unnecessary

Authors: It has now been removed.

370 I would soften this statement even more, given that there are no data to suggest this elevational pattern.

Authors: We have rephrased this to show that this may be a plausible hypothesis.

404 What does "less integrated" WUE mean? d13C is often used as an "integrated measure of WUE" but there is no more or less integrated in this context.
Authors: This has now been corrected, we meant less conservative water use.

407 One of the most iconic examples of sexual dimorphism in plants is the genus Leucadendron (Proteaceae) that occurs in the Cape Floristic Region. Harris and Pannell (2010, J. Ecology) performed a rigorous phylogenetic test of whether maternal investment (their theory was based in plant hydraulic architecture) drives the degree of dimorphism in the genus, and they reported support for this. However, Midgley et al (2017), Midgley and Cramer (2022 Scientific Reports), and Roddy et al. (2019 PeerJ) have argued (fairly convincingly) to the contrary. (Admittedly, I am a bit biased by this latter argument!)
Authors: We have added the suggested references to include the contrasting evidence.

479 I'm confused by the remarkable nature of this finding. The wording "almost specifically" is a little confusing-do you mean that such large a lag is expected only in arid habitats?. Is it that the 30-month lag is higher than expected?

Authors: Yes, thanks for this appreciation. We did not expect a very large delay in the growth response of *I. aquifolium* to SPEI, which was only observed in tree species growing in semiarid areas. We have reformulated this sentence: “These extremely delayed drought legacies can be observed in trees growing in arid or semiarid environments under chronic water deficit”.

491 Correct formatting of this reference.

Authors: Done.

496 Yes, the costs of reproduction are critical to know. Are there data on the different costs of reproduction for males versus females, either at the level of individual reproductive organs or the whole plant?
Authors: We have added information regarding costs of reproduction in *I. aquifolium*, in particular: ‘adult female trees of this species usually incur higher costs of reproduction than adult male trees’.

524 I don't disagree with this interpretation of the statistical results. But I'm wondering if this makes sense biologically. Why (or how) would such a long lag be experienced by the plant? Could this be related to the costs of reproduction in any one year? As far as I can tell, there is no accounting for the interannual variation in reproductive output; reproduction in one year can certainly affect reproductive output in subsequent years. So if water is limiting, perhaps the females invest a lot in reproduction (perhaps to maximize reproduction if prolonged drought is coming), but that does come at the cost of some future growth or reproduction.

Authors: We agree with this argument. However, the obtained correlation shows that the association between female growth and 30-month SPEI is continuous over the period 1942-2001. This means that not only water availability is limited in certain years and reproduction is maximum in certain years to avoid these limiting conditions, but also that the limitation is permanent. As we argued in the Discussion, we believe that the most plausible explanation should be sex-specific functional traits related to sexual reproduction that result in less efficient water use in females than in males. This means that females have high carbohydrate requirements due to their high reproductive costs, and that females then require a longer period of sufficient water availability to maintain high rates of photosynthesis than males. In contrast, males require fewer carbohydrates for reproduction and a shorter period of good water availability may be sufficient to produce the carbohydrates required for reproduction and growth.

In the light of the Handling Editor's views given above I am pleased to say that your paper can be conditionally accepted subject to a satisfactory revision being sent to us within four weeks.

**Reviewer Comments:**

Referee's Review for 24204:

Comments for the Author (Required):

Title: Differential growth rate, water use efficiency and climate sensitivity between male and females Ilex aquifolium in north-western Spain

Authors: Sánchez Vilas et al

This paper describes a tree ring study to disentangle sex-specific responses to climate over many decades in a widely distributed tree species, European holly. Using analysis of basal area increment and carbon isotope ratios in wood cellulose, the authors show a strong age by sex response to climate conditions. Overall, this is a very nice paper and adds to the body of evidence of sexual dimorphism in long-lived trees. I have several minor comments below that should be considered by the authors. My main comment is that some of the important climate variables over time are not well reported despite their importance in modeling sex-specific patterns of growth and photosynthetic habit over time. An added figure of annual mean annual temperature, or mean annual potential evapotranspiration would help the interpretation of the growth trends over time.

Specific comments.

Lines 8 and 9: Change to "stable carbon isotope abundance of wood cellulose".
Authors: Done

Line 12: Not sure what is meant by "unstable". Maybe change to "variable".
Authors: Done

Line 14: Change "next" to "following".

Authors: Done

Line 15: Change "last" to "the previous"

Authors: Done

Line 81 and throughout: Change carbon stable isotopes to stable carbon isotopes.
Authors: Done

Lines 81-87: Incomplete sentence

Authors: This has now been corrected.

Line 99: some added context of why European holly was chosen for study.
Authors: This has now been added in the material and methods.

Lines 102-103: I don't understand what is meant by "prove a sex-dependent response". Please re-phrase.

Authors: This has now been rephrased.

Line 158: What is a semi-automated device? Be more specific.

Authors: We have now added details on the device used as follows: “the annual ring widths were measured to the nearest 0.01 mm using a dendrochronometer (digital positiometer, Kütschenreiter, Austria)”.

Lines 180-181: this is a bit confusing because here you say that 13C was measured on the bulked wood material, but what you describe is the extraction of holocellulose. This needs some clarification.

Authors: Yes, we acknowledge that this may be a bit confusing.

What we meant by ‘bulked material’ is that we measured carbon isotope composition in groups formed by five ring sequences (pentads), i.e. the five rings were bulked together, then ground and the carbon isotopic composition measured on the bulk sample of these five rings.

We have now changed the sentence to avoid this confusion (lines 187-190 in the revised manuscript).

Lines 280-282: Were the differences in BAI statistically significant? Either way, need to come clean on the stats.

Authors: Here, we start the paragraph describing the overall mean values, but also highlighting the different trends of males and females through time as indicated in figures 2 A and 2B; these different trends are then corroborated by the stats that show a significant interaction between sex and time (pentad). We have now rephrased the text here to clarify this.

Lines 320-336: Is it likely that the differences between sexes in environmental sensitivity is related to differential timing in sink strength as a function of different reproductive phenologies? The differential timing in flowering phenology is discussed on 387, but circle back to what the potential drivers are for the differences in sensitivity among the sexes.

Authors: Thanks for the suggestion. We have now added a sentence in the Discussion to point to the differential timing in sink strength as a potential driver of different sensitivity among the sexes (lines 487-490 in the revised version).

Lines 366-367: And females often grow at a higher rate in high moisture environments (Ward et al., 2002, Tree Physiology 22: 339-346).

Authors: This has been added here.

Lines 396-401: Alternative hypothesis might be changes in climate is driving larger reductions in female growth relative to males. I would recommend adding a figure of annual temperature, or PET (not just the means shown in Fig 1) over the period ring widths were measured.

Authors: Yes, since females are more affected by increased temperatures than males, a rising temperature trend would impact females more than males. We have added a new graph to Fig. 1 showing the temporal variation of mean annual temperature in the 20^th^ century and its linear fit. We have included this result on lines 151-152 and this reasoning in lines 408-412 in the revised manuscript.

Lines 404-405: You mean "less conservative water use than males".

Authors: This has been corrected.

Line 414: Good idea to cite Dawson and Ehleringer, 1993; and Hultine et al., 2013 here as well.

Authors: Done.

Lines 416-418: Again, could the changes in relative growth between sexes be related to changes in PET / temperature in the latter half of the 20th century relative to the first half?

Authors: We have added a new graph to Fig. 1 showing the temporal variation of mean annual temperature in the 20^th^ century and its linear fit. We have added this result on lines 151-152 in the revised manuscript.

Lines 441-474: Excellent points, and covers many of the points I brought up above. Illustrates why it would help to provide more information about the climate conditions year over year, in a separate graph.

Authors: Thank you for your positive appreciation.

Authors: In addition to these changes, we have added a new reference of the *olsrr* software:

Hebbali A. 2020. *olsrr*: Tools for Building OLS Regression Models. R package version 0.5.3. <https://CRAN.R-project.org/package=olsrr>
